# Three-dimensional hepatocyte culture system for the study of *Echinococcus multilocularis* larval development

**DOI:** 10.1371/journal.pntd.0006309

**Published:** 2018-03-14

**Authors:** Li Li, Bing Chen, Hongbin Yan, Yannan Zhao, Zhongzi Lou, Jianqiu Li, Baoquan Fu, Xingquan Zhu, Donald P. McManus, Jianwu Dai, Wanzhong Jia

**Affiliations:** 1 State Key Laboratory of Veterinary Etiological Biology, Key Laboratory of Veterinary Parasitology of Gansu Province, Lanzhou Veterinary Research Institute, Chinese Academy of Agricultural Sciences, Lanzhou, Gansu, P. R. China; 2 State Key Laboratory of Molecular Developmental Biology, Institute of Genetics and Developmental Biology, Chinese Academy of Sciences, Beijing, P. R. China; 3 Molecular Parasitology Laboratory, QIMR Berghofer Medical Research Institute, Brisbane, QLD, Australia; University of Buenos Aires, ARGENTINA

## Abstract

**Background:**

Hepatocyte-based metacestode culture is an attractive method to study alveolar echinococcosis (AE), but it is limited by the relatively short lifespan of cultured hepatocytes in maintaining their normal function.

**Methodology/principal findings:**

We describe a three-dimensional (3D) hepatic culture system developed from co-cultured hepatocytes and mesenchymal stem cells using a collagen scaffold to study the development of *Echinococcus multilocularis* larvae. This 3D culture system preserved the function of hepatocytes for a longer period of time than their monolayer counterparts, with albumin secretion, 7-ethoxyresorufin O-deethylation activity, urea synthesis, CYP3A4 and CYP2D6 activity being highly preserved for 21–28 days. The expression levels of hepatocyte-specific genes including CLDN-3, Bsep, AFP, G6P, A1AT, CYP3A4 and NR1I3 were significantly higher in the 3D cultured system compared with their monolayer counterparts after 14-days in culture. Additionally, in the presence of 3D cultured hepatocytes, 81.2% of *E*. *multilocularis* protoscoleces rapidly de-differentiated into infective vesicles within eight weeks. Transcriptomic analyses revealed 807 differentially expressed genes between cultured vesicles and protoscoleces, including 119 genes uniquely expressed in protoscoleces, and 242 genes uniquely expressed in vesicles. These differentially expressed genes were mainly involved in parasite growth relating to the G-protein coupled receptor activity pathway, substrate-specific transmembrane transporter activity, cell-cell adhesion process, and potentially with neuroactive ligand-receptor interaction.

**Conclusions/significance:**

This culture system provides a valuable advance in prolonging hepatocyte functionality, a foundation for future in-depth analysis of the host-parasite interaction in AE, and a useful model to evaluate potential therapeutic strategies to treat AE.

## Author summary

Alveolar echinococcosis (AE) is one of the world’s most dangerous zoonoses. Although there have been recent advances in some aspects of the molecular biology of *E*. *multilocularis*, larval development is far from understood. An *in vitro* hepatocyte based cultivation system for the metacestode stage of *E*. *multilocularis* has been developed to improve our understanding of AE. However, in two-dimensional conventional cultures, hepatocytes rapidly lose key phenotypic and functional characteristics after only approximately seven days. This hinders long-term *in vitro* studies of *E*. *multilocularis* larvae, which require several months for development. Thus, in this paper, a three-dimensional (3D) hepatic model was developed for simulating the organotropism of *E*. *multilocularis* toward the liver of its intermediate host. This 3D model can preserve the functions of hepatocytes and results in rapidly developed *E*. *multilocularis* larva. Genes uniquely expressed in protoscoleces and vesicles provided key information for the further study of AE. The 3D hepatic model provides a new foundation for *E*. *multilocularis* developmental studies and in-depth analysis of the host-parasite interaction in AE.

## Introduction

Human alveolar echinococcosis (AE), is caused by the larval stage of the tapeworm *Echinococcus multiloculari*s, one of the most lethal of the human helminthiases. Untreated AE has a fatality rate of >90% in humans, causing great public health concern in the northern hemisphere [1,2]. China is believed to account for the majority of global AE cases, where 230,000 individuals presently suffer from the disease with a total population at risk of some 22.6 million in 7 provinces, representing nearly 50% of China’s total area [1,3]. Human infection, following the ingestion of *E*. *multilocularis* eggs, results in the development of metacestodes in the liver with clinical signs such as an abdominal mass and/or pain, jaundice and, ultimately, liver failure [3]. The metacestode tissue proliferates and infiltrates host tissue like a malignant tumour, eventually giving rise to numerous protoscoleces that either develop into the adult stage, when transmitted to the definitive host, or ‘de-differentiate’ towards the metacestode, when distributed in the intermediate host [4,5]. Understanding the biological mechanisms of *E*. *multilocularis* metacestode development and differentiation is the key for the control and treatment of AE.

Recently, several *in vitro* cultivation systems, including an axenic (host cell-free) cultivation system and a co-cultivation system for the metacestode stage of *E*. *multilocularis* have been developed to improve our understanding of AE [6–8]. These systems rely on the incubation of small tissue blocks, or vesicle suspensions from an infected host in a conditioned medium prepared based on feeder cells, or with co-cultured feeder cells in a medium. In essence, the feeder cells, including immortalized cell lines and primary cultured hepatocytes from rats or humans, support the culture of the parasite and provide good conditions for vesicle development. In particular, the hepatocyte based co-culture system showed good potential for studies on host-parasite interactions in AE [9].

However, in two-dimensional (2D) conventional cultures, hepatocytes develop into physiologically compromised cells and have the major drawback of the rapid loss of key phenotypic and functional characteristics after approximately seven days [10,11]. The dedifferentiation of hepatocytes even initiated during the first 24 hours of 2D culture[12]. Although various growth factors and other important constituents are supplied in the medium in 2D hepatocyte cultures, in the absence of a natural matrix-like environment, in which many such proteins are present in a bound state, normal growth and differentiation do not occur[13,14]. The spontaneous dedifferentiation of hepatocytes in 2D culture hinders long-term *in vitro* studies. This is particularly true for *in vitro* study of *E*. *multilocularis*, which grows slowly and requires a long period (several months) for the development of the life cycle. Thus, a novel culture system that allows extended hepatocyte maintenance and long-term *E*. *multilocularis in vitro* studies is required.

Currently it is well recognized that the functionality of hepatocytes can be improved and extended with time when they are cultured as 3D-cell aggregates [15–17]. Hepatic culture in 3D has been shown to change gene expression, cell phenotype and cell surface receptor expression towards more liver-like properties [18,19]. Moreover, there has been a shift in the study of infectious-disease mechanisms from the use of 2D monolayers to the use of organotypic or 3D culture models that mimic the morphological and functional features of their *in vivo* parental tissues. For example, a 3D liver model was shown to mimic the differentiated and polarized state of hepatocytes *in vivo* more closely than a monolayer, and this model was also shown to be highly permissive for infection with hepatitis C virus, for which robust cell culture and small-animal infection models had been used in the past [20]. For this pathogen, a physiological *in vivo*-like 3D cell culture model has provided an enabling tool for researchers to investigate those host-pathogen interactions *in vitro*, which previously were difficult or impossible to study [20, 21]. 3D models have been used to study *Entamoeba histolytica* and *Cryptosporidium parvum* which require only a few days for proliferation [22,23]. Studies using 3D models on larger multicellular parasites, such as *E*. *multilocularis*, which can be maintained only in a specific host or under specific culture conditions and develop over the course of several months, are lacking.

In this paper, we describe a 3D hepatic model that we developed from co-cultured hepatocytes and mesenchymal stem cells (MSCs) based on a collagen scaffold. We used this 3D hepatic model to establish an *E*. *multilocularis* infection system to study the growth and development of protoscoleces and vesicles to gain insight into the molecular mechanisms that mediate protoscolex development. This research provides new insight for study of the development of *E*. *multilocularis* larvae and offers a new approach for the control and treatment of echinococcosis.

## Materials and methods

### Ethical statement

All experiments involving mice were performed under strict Chinese experimental animal clearances, and mice at all times were treated in agreement with animal ethics procedures and guidelines for animal husbandry of the Institutional Ethics Committee of Lanzhou Veterinary Research Institute, Chinese Academy of Agricultural Sciences. The study and the use of mice were approved by this Committee (Approval No. LVRIAEC2010-005). In addition, all mice were handled in strict accordance with the animal protection laws of the People’s Republic of China (A Draft of an Animal Protection Law in China released on September 18, 2009).

### Materials

All chemicals were purchased from Sigma Aldrich (St. Louis, MO, USA) if not specifically mentioned. Dulbecco’s modified Eagle’s medium (DMEM), penicillin-streptomycin, Dulbecco’s phosphate buffered saline (D-PBS), and fetal bovine serum (FBS) were purchased from Hyclone (GE Healthcare Life Science, Logan, Utah, USA). Albumin ELISA kits were purchased from TSZ Biosciences (USA TSZ Biological Trade Co., Ltd., USA). Kits for EROD ELISA were purchased from Shanghai Bluegene Biotech Co., Ltd. (Shanghai, China) and QuantiChrom urea assay kits were purchased from Bioassay Systems (Hayward, USA). P450-Glo CYP3A4 assay (Luciferin-IPA) kits and CYP2D6 assay (Luciferin-ME EGE) kits were purchased from Promega Corporation (Madison, USA). Collagen scaffolds were fabricated from collagen membranes obtained from Zhenghai Biotechnology Ltd. (Shandong, China). Briefly, the collagen membranes were immersed in a 0.5 M acetic acid solution for 8 h at 4°C, then the solution was mixed in a blender for 15 min to obtain a homogeneous collagen solution and neutralized with 4 M NaOH. The homogeneous solution was dialyzed against deionized water for five days and lyophilized. The porous collagen scaffolds obtained were cut into 0.1×0.5×0.5 cm pellets and crosslinked by 1 mg/ml 1-ethyl-3-(3-dimethylaminopropyl) carbodiimide (EDC) and 0.6 mg/ml N-hydroxysuccinimide (NHS) as before[24]. After crosslinking, the pellets were lyophilized again, sterilized with Co60, and stored at 4°C for utilization.

### Cell culture

Hepatocytes and MSCs were isolated from adult male BALB/c mice (20 g; Lanzhou University, Lanzhou, China) [25,26]. For 3D co-cultures of hepatocytes and MSCs [Hepatocytes-MSCs (3D)], the collagen wafer was loaded with the mixed suspension of hepatocytes (5×10^5^ cells) and MSCs (1×10^5^ cells), and maintained in DMEM media supplemented with 10% FBS (v/v), 1 mM sodium pyruvate, 100 U/mL penicillin, 100 g/L streptomycin, 10 μg/L epidermal growth factor, 1% non-essential amino acids (v/v), 0.5 U/mL insulin, 10 M dexamethasone, and 100 mM L-ascorbic acid. The culture plates with scaffolds together with seeded cells were placed on a shaking table, and half of the cell culture media was changed every two days. In addition, three cell culture groups including i) 2D hepatocyte cultures [Hepatocytes (2D)], ii) 2D hepatocyte and MSC co-cultures [Hepatocytes-MSCs (2D)], and iii) 3D hepatocyte cultures [Hepatocytes (3D)] were used as controls. For 2D hepatocyte cultures, freshly harvested hepatocytes were seeded onto culture plates pre-coated with matrigel at a density of 5×10^5^ total cells. For 2D hepatocyte and MSCs co-cultures, freshly harvested hepatocytes and MSCs were simultaneously seeded onto culture plates pre-coated with matrigel at a density of 5×10^5^ cells/cm^2^ and 1×10^5^ cells/cm^2^. After seeding, the cultures were untouched for 24 h at 37°C in a humidified atmosphere with 95% air and 5% CO_2_ to allow cell attachment. Unattached cells were then removed by a medium change. Half of the culture medium was changed every 2 d. For 3D hepatocyte cultures, freshly harvested hepatocytes (5×10^5^ total cells) were seeded onto 3D collagen pellets. The subsequent operating procedure was the same as for the Hepatocytes-MSCs (3D). Culture supernatants and cells in the different groups were collected according to the protocol at the same time points for further assay.

### Cell viability assay

To investigate cell viability and apoptosis, cells cultured on the scaffold were observed by double staining with fluorescein diacetate (FDA) and propidium iodide (PI). A scanning electron microscope (SEM, JSM-6380LV, JEOL, Tokyo, Japan) was used to visualize the surface features of the scaffold and cells cultured in the scaffold. The samples were visualized with the SEM using an accelerating voltage of 30 kv. For detailed information on SEM sample preparation, see S1 Text. For Immunocytochemistry, the cultures were prepared as previously described [27]. The primary antibodies used were as follows: polyclonal rabbit anti-cytokeratin 8 antibody (CK8, 1:100, ab137855, Abcam) and monoclonal rat anti-CD44 antibody (1:100, ab119348, Abcam). Then, followed by secondary goat anti-mouse IgG-TRITC (1:1000, ab6786, Abcam), or a goat anti-rabbit IgG-FITC (1:1000, ab7086, Abcam) antibody.

### Measurement of differentiated hepatocyte functions

Albumin secretion (production) of the cultured hepatocytes was examined daily with a murine albumin ELISA quantitation kit. Urea synthesis by cultured hepatocytes incubated in culture medium with 2 mM NH_4_Cl for 4 h was measured with a QuantiChrom urea assay kit according to the manufacturer’s instructions. The EROD assay was initiated by incubating the hepatocytes with 39.2 μM 7-ethoxyresorufin in the culture medium at 37°C for 4 h. The amount of resorufin converted by the enzyme was determined with EROD ELISA kits. The albumin, urea, and EROD quantifications were performed in triplicate.

### Determining cytochrome P450 enzyme activity

Cytochrome P450 (CYP450) enzyme activity, including CYP3A4 and CYP2D6 activity was monitored using Promega-Glo assays (Promega Corp., Madison, USA,). Briefly, the induction reagent (500 μM of phenobarbitol for CYP3A4 and CYP2D6 induction) was diluted in medium and added to the hepatocytes culture plates for 48 h. We then washed the reservoirs with buffer three times for 5 min and subsequently replaced the buffer with luciferin-IPA and luciferin-EGE, respectively. At the end of the incubation period, medium was collected and transferred into 96-well plates (white). Detection reagent was added to each well, and luminescence was read with a Veritas Microplate Luminometer using the settings provided by the manufacturer. Results were expressed as multiples of the level of induction observed in controls that were not treated with the induction reagent.

### RT-PCR

Total RNA extraction from cultured cells was undertaken using an RNeasy kit, and reverse transcripts were synthesized using a RevertAid First Strand cDNA Synthesis kit (Thermo Fisher Scientific Inc.). PCR was performed in a 20-μL reaction containing 2 μL of cDNA as a template with each specific oligonucleotide primer pair, with GAPDH used as an internal control. The mRNA levels of claudin-3 (CLDN-3), bile salt export pump (Bsep), alpha-fetoprotein (AFP), glucose-6-phosphatase (G6P), α-1 antitrypsin (A1AT), CYP3A4, and nuclear receptor subfamily 1, Group I, member 3(NR1I3) were determined. See S1 Text for details on the primer sequences and PCR procedures used. Quantitative analysis of the bands obtained in the PCR was performed using Image-pro plus (Media Cybernetics, Inc., USA).

### Cultivation of protoscoleces

Larval material was obtained from BALB/c mice experimentally infected with *E*. *multilocularis* homogenized larval tissue, which was originally isolated from a naturally infected plateau pika (*Ochotona curzoniae*) collected in Yushu, Qinghai province, China. For detailed information on the preparation of protoscoleces and molecular identification of the *E*. *multilocularis* isolate, see S1 Text and S10 Fig. For 3D culture, the collected protoscoleces were re-suspended in DMEM media and 50 μL of this suspension containing 200 protoscoleces was then added to the culture dishes of the 3D cell cultures at day 3; half of the cell culture media was changed every two days. After culturing for four weeks, a new collagen scaffold, with the 3D co-culture of the hepatocytes and MSCs for three days, was added to the culture dishes with the protoscoleces. For 2D culture, 50 μL of DMEM media containing 200 protoscoleces was added to the dishes of the 2D hepatocytes and MSCs co-cultured at day 3; half of the cell culture media was changed every two days. After culturing for four weeks, the cultured protoscoleces was moved to new culture dishes with 2D hepatocytes and MSCs co-cultured at day 3. Meanwhile, 50 μL of the suspension containing 200 protoscoleces and 200 μL of culture medium was added to the culture dishes with collagen scaffold (without cells) as control; half of the cell culture media was changed every two days. After culturing for four weeks, a new collagen scaffold, was added to the culture dishes with the protoscoleces. Protoscoleces development and vesicles growth were monitored, and images were captured every 24 h. The viability of the protoscoleces was detected by 0.1% trypan blue exclusion test. For RNA isolation, protoscoleces were incubated in DMEM media for 24 h at 37°C and 5% CO_2_.

### Experimental infection of the cultured vesicles

Forty-eight female BALB/c mice (25 g, Lanzhou University, Lanzhou, China) were housed in a temperature-controlled light cycled room with food and water *ad libitum*. *In vitro* cultured vesicles that reached a diameter of more than 3 mm were collected and macerated. Then the tissue and vesicle fluid were separated by centrifugation. Experimental infection was carried out by intraperitoneal injection of the sedimented material from 50 vesicles per mouse. We also injected protoscoleces intraperitoneally into BALB/c mice as a control (50 protoscoleces per mouse). The animals were euthanized after one, two, and three months, and then necroscopy was performed. The parasite material was collected and fixed in 10% (v/v) formalin and then embedded in paraffin for 4-μm sections. Hematoxylin and eosin (H&E) staining was used for morphologic analysis.

### Parasite RNA-seq

Approximately 1,000 protoscoleces and 50 vesicles (the vesicles were cultured for eight weeks and reached a diameter of more than 5 mm) were used for total RNA extraction. TRIzol reagent (Invitrogen, Carlsbad, CA) was used to extract the total RNA from these samples. RNA integrity was assessed using the RNA Nano 6000 Assay Kit of the Bioanalyzer 2100 system (Agilent Technologies, CA, USA). Sequencing libraries were generated using NEB Next Ultra RNA Library Prep Kit for Illumina (NEB, USA) and index codes were added to attribute sequences to each sample. Full details on RNA library preparation are provided in the S1 Text. All downstream analyses were based on clean data of high quality. HTSeq v0.6.1 was used to count the read numbers mapped to each gene. Then, the RPKM of each gene was calculated based on the length of the gene and the reads count mapped to this gene.

Differential expression analysis of two groups (three biological replicates per condition) was performed using the DESeq R package (1.10.1). Genes with an adjusted P-value <0.05 by DESeq were assigned as being differentially expressed. Gene ontology (GO) enrichment analysis of differentially expressed genes was implemented using the GOseq R package in which gene length bias was corrected. GO terms with corrected P-values less than 0.05 were considered significantly enriched by differentially expressed genes. KOBAS software was used to test the statistical enrichment of differentially expressed genes in metabolic KEGG (Kyoto Encyclopedia of Genes and Genomes) pathway analysis.

### qPCR

To validate the repeatability and reproducibility of the gene expression data obtained by sequencing of RNA from cultured vesicles and protoscoleces, qRT-PCR was carried out on 40 randomly selected differentially expressed genes. For detailed information on the procedures of qRT-PCR, see S1 Text.

### Image acquisition and analysis

3D cultures were subjected to confocal microscopy (Leica TCS SP8, Germany). Bright field and fluorescence images of 2D cultures were acquired using an inverted microscope (Olympus, IX71) equipped with a CCD camera (Olympus, DP71) and a mercury lamp (Olympus, U-LH100HG). DP Controller software, DP Manager (Olympus Corporation) and SPSS 12.0 (SPSS Inc.) were employed for image acquisition and statistical data analysis, respectively. Results, including graph error bars are presented as the mean ± S.D. Tests of data significance were performed using one-way ANOVA. The confidence level was set at 0.05 for all tests with p-values less than 0.05 being considered statistically significant.

## Results

### Cell morphology and viability assay

**Fig 1:**
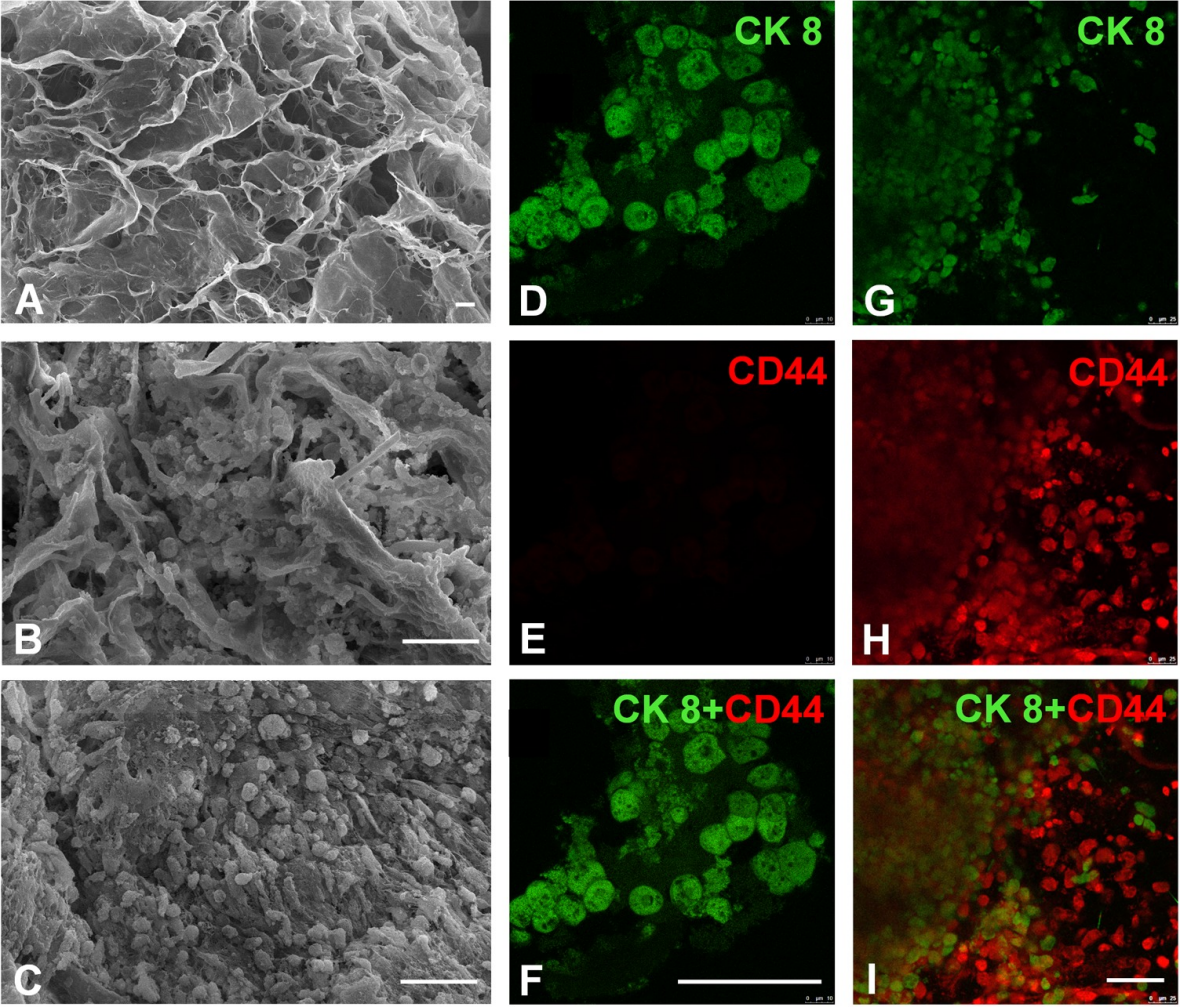
SEM images of a 3D collagen scaffold (A), hepatocytes cultured for 21 d in the scaffold (B), and hepatocytes and MSCs co-cultured for 21 d in the scaffold (C). Fluorescent images of immunocytochemical staining of hepatocytes cultured for 21 d in the scaffold (D-F), and hepatocytes and MSCs co-cultured for 21 d in the scaffold (G-I). Scale bar: 50 μm.

**Fig 2:**
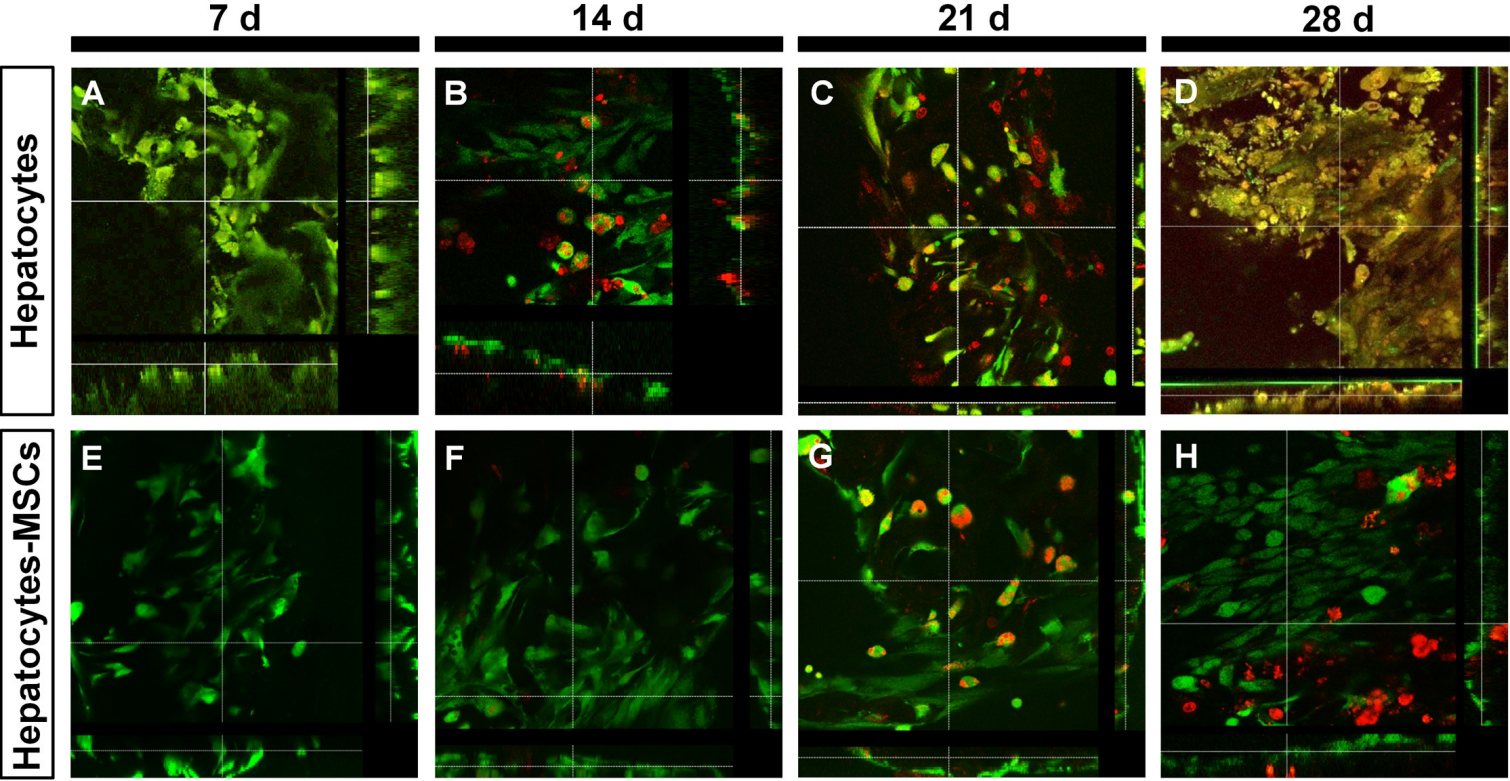
Viability assay of 3D cultured cells. (A-D) A set of FDA-PI double-staining fluorescent images of cells in Hepatocytes (3D) groups after culture for 7, 14, 21, and 28 days. (E-H) A set of FDA-PI double-staining fluorescent images of cells in Hepatocytes-MSCs (3D) groups after culture for 7, 14, 21, and 28 days. Live cells are stained green, and dead cells are stained red.

Cells were cultured in 1 mm thick, 5 mm diameter porous collagen scaffold wafers (S1 Fig). The scaffold maintained the porous structure with non-uniform apparent micro-holes from 40 μm to 600 μm in diameter (Fig 1A). After culturing for 21 d, the upper layers of cells in the Hepatocytes (3D) group were unevenly distributed over the pores of the scaffold (Fig 1B). In contrast, the upper layers of cells in the Hepatocytes-MSCs (3D) groups were equally distributed over the scaffold area forming a dense; the holes of the scaffold were full of cells and the structure of the scaffold was barely visible (Fig 1C). Immunocytochemistry showed that the cells of the Hepatocytes (3D) group were only CK8-positive and exhibited a cuboidal shape, confirming the group was composed of hepatocytes (Fig 1D–1F). The cells within the Hepatocytes-MSCs (3D) tissue were CK8-positive (stained green) or CD44-positive (stained red), confirming that the 3D tissue was predominately composed of hepatocytes and MSCs (Fig 1G–1I). Cell viability was similar in these two groups during culture for seven days (Fig 2). However, after culturing for 14–28 days, the viability of the Hepatocytes-MSCs (3D) group was higher than the Hepatocytes (3D) group. We observed a difference in cell numbers between these two groups because of the proliferation of the MSCs. These results showed that the MSCs in the 3D organization might enhance hepatocyte viability compared with the control group. In the 2D hepatocytes and MSC cultures, these cells exhibited typical morphology and were immuno-positive for their specific surface biomarkers CK8 and CD44, respectively (S2 Fig).

### Determination of the specific functions of hepatocytes

It was critically important to assess whether the 3D collagen scaffold-combined MSC co-culture could facilitate and maintain the specific functions of hepatocytes *in vitro*. Albumin production, urea synthesis, and 7-ethoxyresorufin O-deethylation (EROD) activity was measured to demonstrate the maintenance of hepatocyte-specific functions in the 3D hepatocyte and MSC co-culture system.

**Fig 3:**
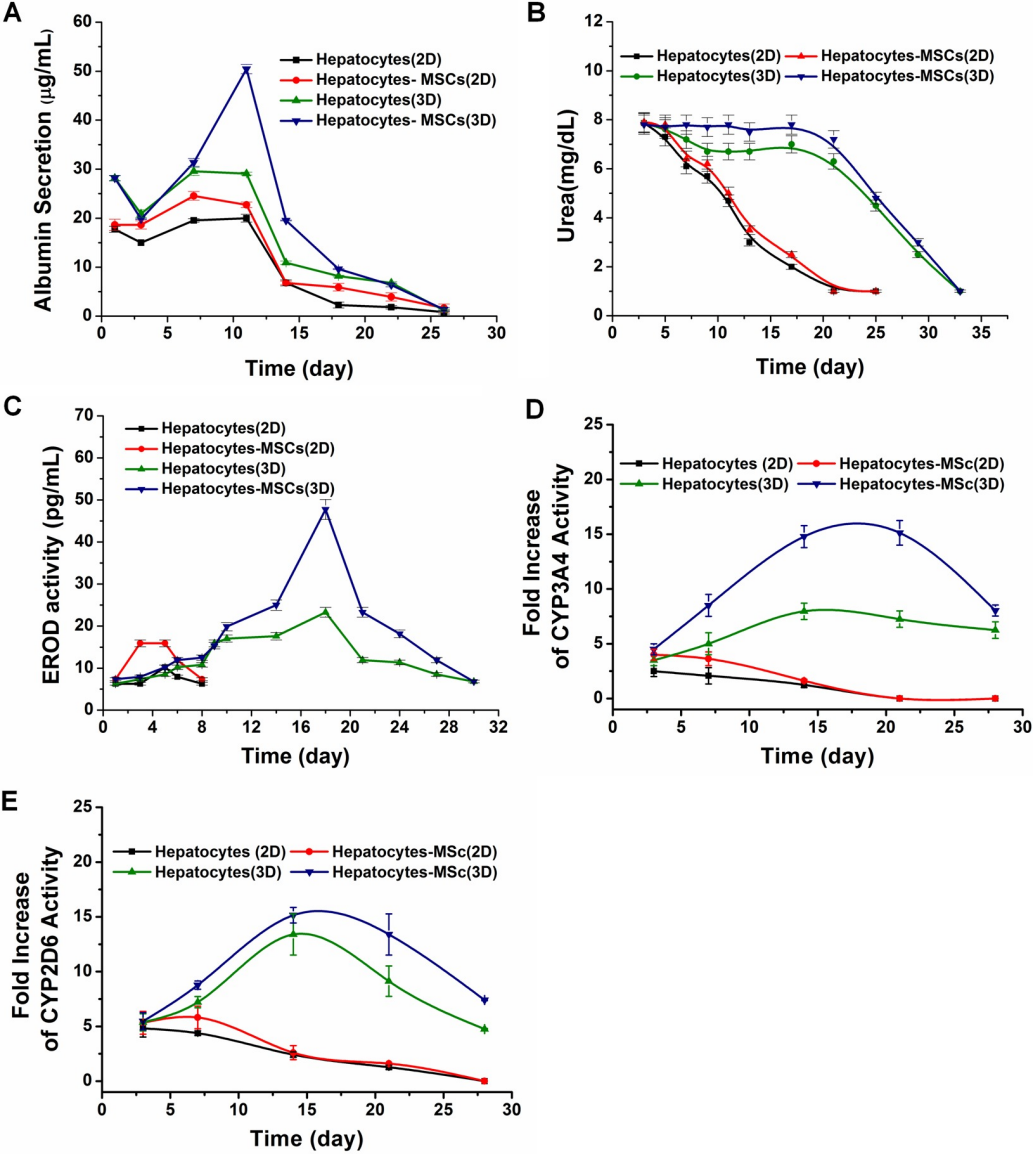
Analysis of hepatocyte function. Hepatocyte functional maintenance at different times as represented by albumin secretion (A), urea production (B), EROD activity (C) CYP3A4 enzyme activity (D) and CYP2D6 enzyme activity (E) relative to the Hepatocytes (2D), Hepatocytes (3D), Hepatocytes-MSCs (2D), and Hepatocytes-MSCs (3D) groups. Activity of CYP3A4 and CYP2D6 enzymes values are shown as fold increase compared to un-induced cultures. Values are means ± standard deviations (S.D.), n = 3 with each separate experiment consisting of two technical replicates.

ELISA analysis showed that the cumulative albumin content in the media of the Hepatocytes-MSCs (3D) group increased remarkably during days 3–11 after culturing commenced (Fig 3A). Albumin production peaked on day 11 and then decreased slightly until day 27. In contrast, the albumin content in the media of the Hepatocytes (2D), Hepatocytes-MSCs (2D) and Hepatocytes (3D) cell culture groups exhibited the same trend, although the level of albumin production in the media was significantly lower at every time point after day 5 compared with the Hepatocytes-MSCs (3D) group. The urea synthesis rate, another index of hepatocyte-specific function, was monitored over 33 days in 3D cultures and 25 days in 2D cultures and significant differences occurred between them (Fig 3B). Consistent with albumin production, urea synthesis in the Hepatocytes-MSCs (3D) group was also significantly higher than in the other three control groups. The urea synthesis activity in this group was stably maintained for up to three weeks. However, a significant decrease in urea synthesis activity over 2 weeks was observed in the 2D cultures.

The biotransformation capacity of hepatocytes was examined by measuring EROD activity (Fig 3C). The EROD content in the media of the Hepatocytes-MSCs (3D) group increased remarkably during days 1–19 and peaked at day 19, then decreased slightly until day 31. In contrast, the EROD activity in the media of the Hepatocytes (3D) cell culture group showed a similar trend but the level of EROD activity in the media was significantly lower at every time point compared with the Hepatocytes-MSCs (3D) group. However, in the media of the Hepatocytes (2D) and Hepatocytes-MSCs (2D) groups, the EROD activity was only detected within a week, and at a lower concentration.

The drug metabolism function of hepatocytes was assayed by monitoring the cell’s capacity to activate CYP3A4 and CYP2D6 enzymes throughout the 28 days of cell culture. Fig 3 shows that both enzymes of the 3D culture group respond to inducers with a maximum fold change (about 15) compared to un-induced controls (Fig 3D and 3E) during days 14–21, then decreased at day 28. These two enzymes in the Hepatocytes (3D) cell culture group showed a similar trend but the level was lower at every time point compared with the Hepatocytes-MSCs (3D) group. However, CYP3A4 and CYP2D6 enzymes activation of the Hepatocytes (2D) and Hepatocytes-MSCs (2D) groups, were only detected within two weeks, and at a lower fold change.

In general, liver-specific functions were significantly higher in the 3D compared with the 2D cultures, higher in co-cultures than in single type hepatocyte cultures, and maintained hepatic cell function for a longer period of up to 21–31 days while a rapid loss of functionality was observed within 7–14 days with the 2D mono-cultures.

### Analysis of hepatocyte-specific gene expression

**Fig 4:**
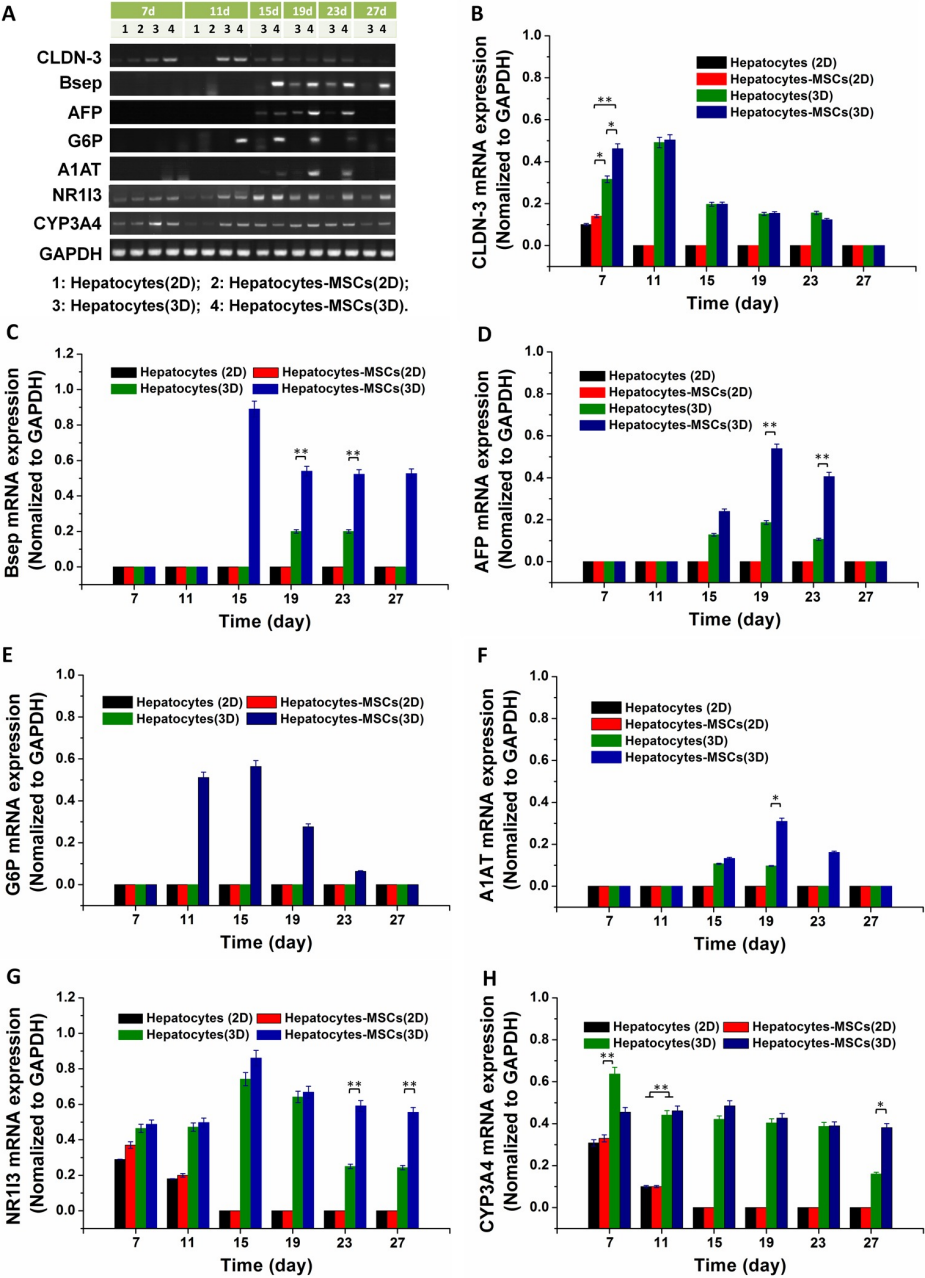
RT-PCR analysis of hepatocyte functional genes. (A) RT-PCR analysis of CLDN-3, Bsep, AFP, G6P, A1AT, NR1I3 and CYP3A4 gene expression in cells from different treatment groups, including Hepatocytes (2D), Hepatocytes (3D), Hepatocytes-MSCs (2D), and Hepatocytes-MSCs (3D) groups. -The expression of the CLDN-3 (B), Bsep (C), AFP (D), G6P (E), A1AT (F), NR1I3 (G) and CYP3A4 (H) genes was normalized with the internal control gene GAPDH. Data are presented as the mean ± S.D., n = 4, with significance assessed by ANOVA. **p< 0.001 and *p< 0.01.

RT-PCR was performed to measure the hepatocyte-specific gene expression levels of hepatocytes cultured in the 3D co-culture system. In Fig 4, gene expression analysis of cells in the Hepatocytes-MSCs (3D) group showed higher levels of mature hepatocyte markers, including CLDN-3 (a tight junction protein is known to express at the outline of bile canaliculi in normal liver tissue), Bsep one of the key components of bile acid efflux in the liver), AFP a marker for hepatic regeneration), G6P (a key regulator of glucose metabolism), A1AT (a protease inhibitor that is considered to prevents from protease-mediated tissue destruction), NR1I3 (a key regulator of drug metabolism and excretion), and CYP3A4 (an enzyme involved in metabolism of ∼50% of medications by the P450 system) [28–32]. Although *in vivo* synthesis of AFP is completely suppressed in normal adult hepatocytes, its re-expression is observed in adult hepatocytes during liver regeneration [31]. There was a temporal increase and then a reduction in the expression levels of all of these genes. There was a significantly higher expression level of each gene analyzed at every time point in the Hepatocytes-MSCs (3D) compared with the other three control groups. The expression of the Bsep, AFP, G6P, A1AT, NR1I3 and CYP3A4 genes rarely appeared in 2D cultures over the same time. These results suggest that the 3D cell co-culture system effectively enhanced the gene expression of hepatocyte-specific functions.

### Parasite culture

**Fig 5:**
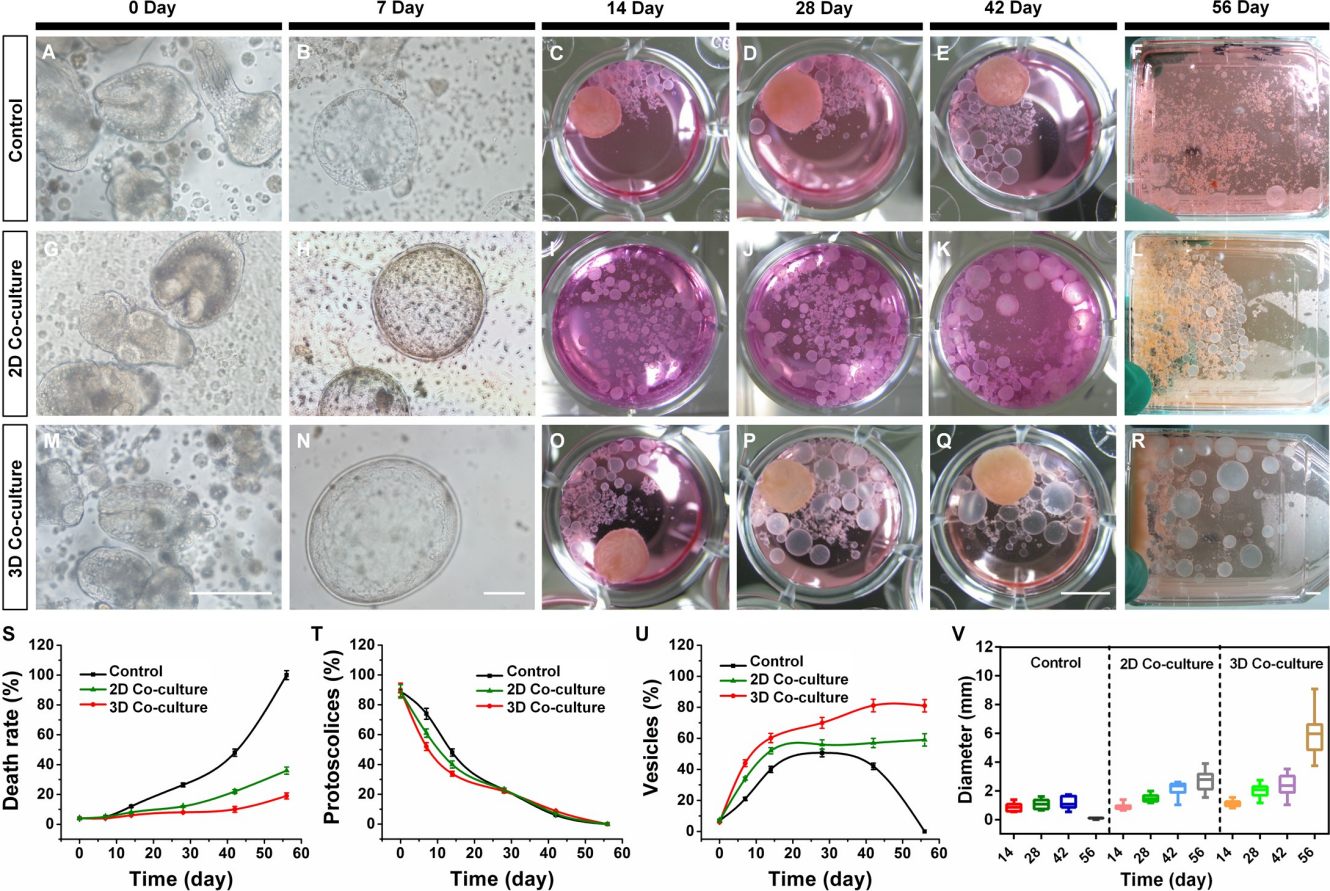
The development of protoscoleces cultured in the control group (A-F), 2D co-cultured group (G-L) and 3D co-cultured group (M-R). (A, G and M) Light microscopic images of invaginated or evaginated protoscoleces cultured for 0 d, scale bar: 100 μm; (B, H and N) De-differentiation and micro vesicles formation of *E*. *multilocularis* protoscoleces after culturing for 7 d, scale bar: 100 μm; (C-F, I-L and O-R) Light images of developed vesicles after culturing for 14, 28, 42, and 56 d, scale bar: 5 mm; The death rate of protoscoleces (S), percentage of protoscoleces (T), percentage of vesicles (U) and diameter of the vesicles (V) in the control group, 2D co-cultured group and the 3D co-cultured group during the culture period.

The Hepatocytes-MSCs (3D) co-culture system was used to study the development of *E*. *multilocularis* protoscoleces. We observed protoscoleces that had evaginated and had become highly motile in the first days of culture (Figs 5A, 5G, 5M and S4). After culturing for one week, the protoscoleces de-differentiated into micro vesicles in the control cultures. The vesicles maintained without hepatocytes-MSCs (3D) showed less of a size increase during culture (Fig 5B–5E); the maximum size of the *E*. *multilocularis* vesicles increased only fourfold from a mean diameter of 0.31 mm to 1.32 mm. It should be emphasized that the sizes of the different vesicles varied considerably in long-term cultures and that vesicles of sizes between 0.12 and 1.66 mm were found after four weeks (Fig 5D–5V). These vesicles appeared healthy during the initial month of culturing. However, by day 42, some vesicles started to show signs of death, with the germinal layer becoming darker and pitted, indicating partial detachment from the laminated layer (S4 Fig, panel C). The degradation continued until 56 d when all of the vesicles showed signs of death (Fig 5F).

In contrast, after culturing for one week, protoscoleces de-differentiated into small vesicles of greater than 100-μm diameter (Fig 5H–5N) in the presence of the 2D and 3D hepatocytes-MSCs. These vesicles grew quickly, but not synchronously, during the following seven weeks (Fig 5I–5L and 5O–5R). In addition, the growth of vesicles was significantly faster in the 3D compared with the 2D co-cultures. In 2D co-culture group, the maximum size of the unilocular vesicles increased about fourfold from a mean diameter of 0.62 mm to 2.71 mm (Fig 5V), and the maximum size of the vesicles reached approximately 4.09 mm. In 3D co-culture group, The maximum size of the unilocular vesicles increased about tenfold from a mean diameter of 0.63 mm to 6.12 mm (Fig 5V), and the maximum size of the vesicles reached approximately 9.06 mm. These vesicles had high intra-cystic pressure and the germinal layer was tightly bound to the laminated layer, indicating they were healthy. Besides, different with co-culture model reported by Jura [9], tissue infiltration could not be observed in our culture system.

Mortality analysis showed that the protoscoleces in the 3D co-culture systems had a lower death rate (a maximum of 18.8%) during the culture period (Fig 5S and S3 Fig). The mortality rate of the control group progressively increased up to 100% after 56 days culture. As shown in Fig 5T, after 6 weeks culture some of the protoscoleces remained alive and were invaginated (8.80% in the 3D co-cultured group, 6.2% in the 2D co-cultured group, 6% in the control group). Additionally, in the control group after 4 weeks culture, only 50.80% of the protoscoleces de-differentiated into small vesicles (Fig 5U). Then, under these culture conditions, the vesicles began to degenerate. After day 56, no intact vesicles were present. In the 2D co-culture system, 56.0% of the protoscoleces de-differentiated into small vesicles after 4 weeks culture and remained at 58.7% by day 56. In contrast, in the 3D co-culture system, the number of vesicles greatly increased from 6% at day 0 to 81.2% after six weeks and remained at 81% by day 56. The 3D hepatocyte-MSCs-parasite cultures could be maintained for at least three months, with continuing growth of the parasitic vesicle, by adding new Hepatocytes-MSCs (3D) co-culture cells every 20 days.

### Parasite infectivity

**Fig 6:**
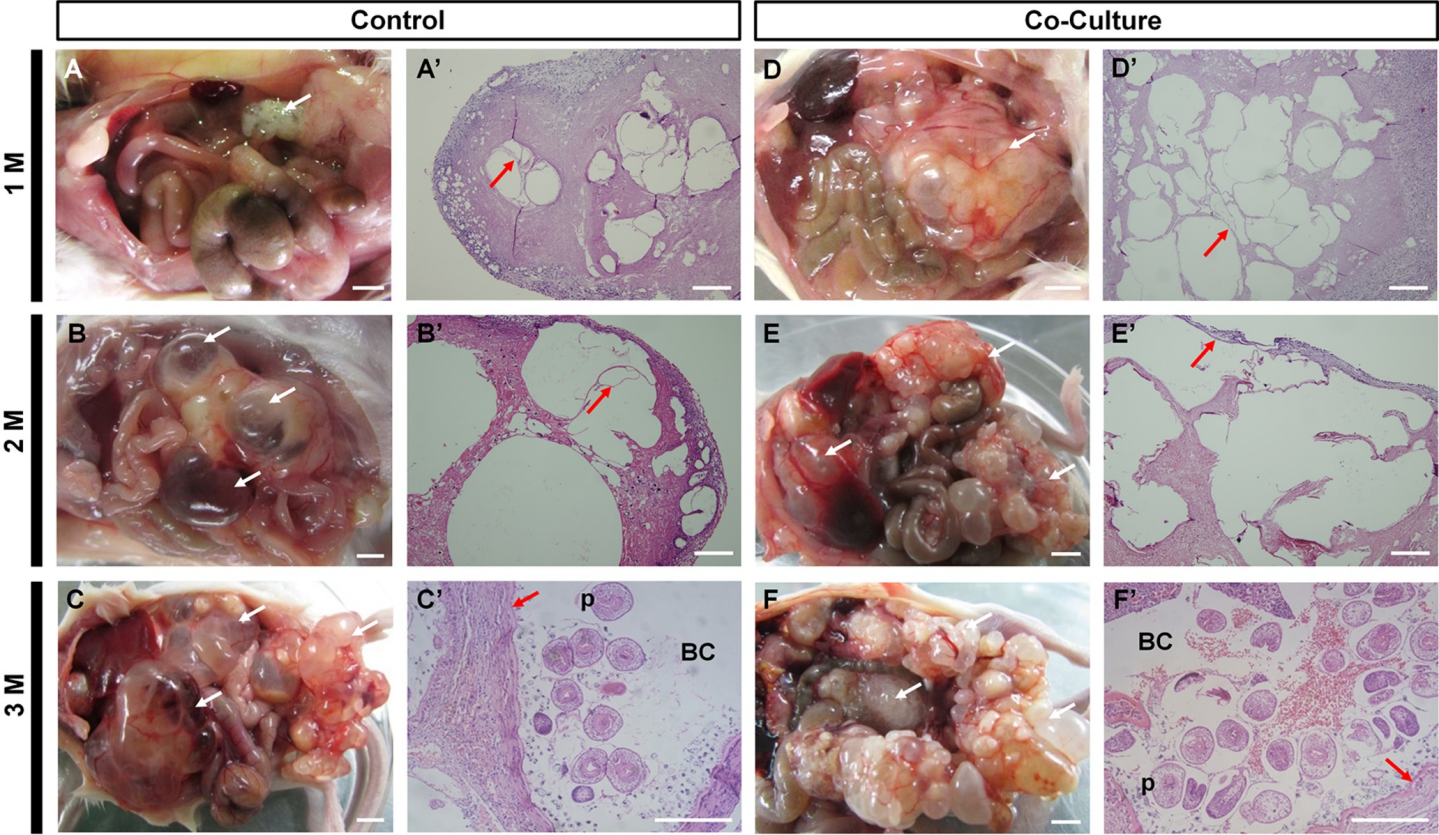
Mouse anatomy after the intraperitoneal injection of protoscoleces (A-C, control group) or vesicles (D-F, co-culture group) after one, two, and three months, scale bar: 5 mm. Microscopic analysis of hematoxylin and eosin staining pictures of metacestode tissue corresponding to A-F, respectively (A’-F’), scale bar: 100 μm. During the proliferative phase, the *E*. *multilocularis* metacestodes (white arrow) emerge from tissue blocks. Hematoxylin and eosin staining shows tissue infiltrates of *E*. *multilocularis* metacestodes, which are composed of fluid-filled microvesicles. The red arrows indicate the laminated layer (LL) of the larvae. The differentiation into protoscoleces (p) commences with the formation of brood capsules (BC) (C’, F’). Compared with the co-culture group, the AE metacestode lesion of the control group is composed of smaller numbers of microvesicles that contain fewer protoscoleces.

Considerable amounts of *E*. *multilocularis* metacestode tissues were detected in the liver, spleen, mesentery, and peritoneum in both groups injected with vesicles and protoscoleces (Fig 6A–6F). The parasite tissues kept growing with increasing size and number of vesicles. The size of the parasite tissue in the vesicle-injected mice was larger than the parasite tissue in the protoscoleces-infected mice. H&E staining showed a regular laminated and germinal layer with numerous well-developed protoscoleces present within the metacestode tissue in both groups of mice (Fig 6A–6F). These results demonstrated that the cultured vesicles had high infectivity. Notably, the parasite loads were significantly higher when *in vitro* cultivated vesicles were used rather than earlier stage protoscoleces.

### Differential gene expression between cultured vesicles and protoscoleces

**Fig 7:**
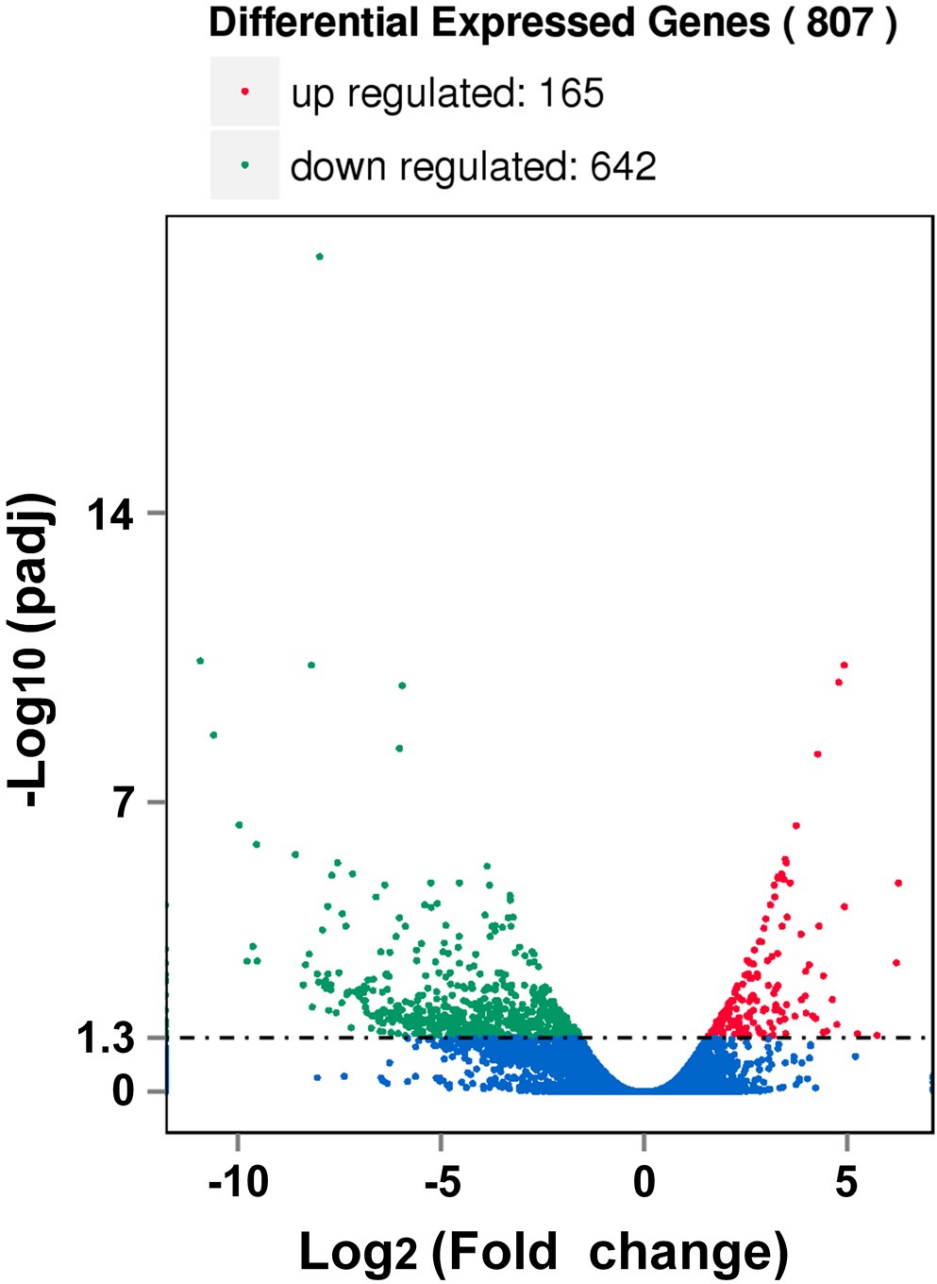
Volcano plot of 807 differentially expressed genes between the cultured vesicles and protoscoleces. Among these, 165 genes were up regulated and 642 were down regulated. The y-axis corresponds to the mean expression value of the log10 (p-value), and the x-axis displays the log two-fold change value. The red dots represent the significantly differentially expressed transcripts (p< 0.05); the blue dots represent the transcripts whose expression levels did not reach statistical significance (p> 0.05) between the vesicles and protoscoleces.

We detected 807 genes as being significantly different between the cultured vesicles (S4 Fig) and protoscoleces, of which, 165 genes were up-regulated while 642 were down-regulated (Fig 7 and S1 Table). We also found 119 genes uniquely expressed in protoscoleces, and 242 genes uniquely expressed in vesicles (S2 Table). These genes were defined while the raw reads count and RPKM of the gene was zero in one stage. We found one gene (EmuJ_000742900.1) up regulated in vesicles compared to protoscoleces. This *Echinococcus*-specific apomucin-encoding gene is important building block of the laminated layer [1]. We also found Biogenic amine 5HT receptor (EmuJ_001171200), a gene putatively involved in serotonin mediated neurotransmission [1], down regulated in vesicles compared to protoscoleces.

**Fig 8:**
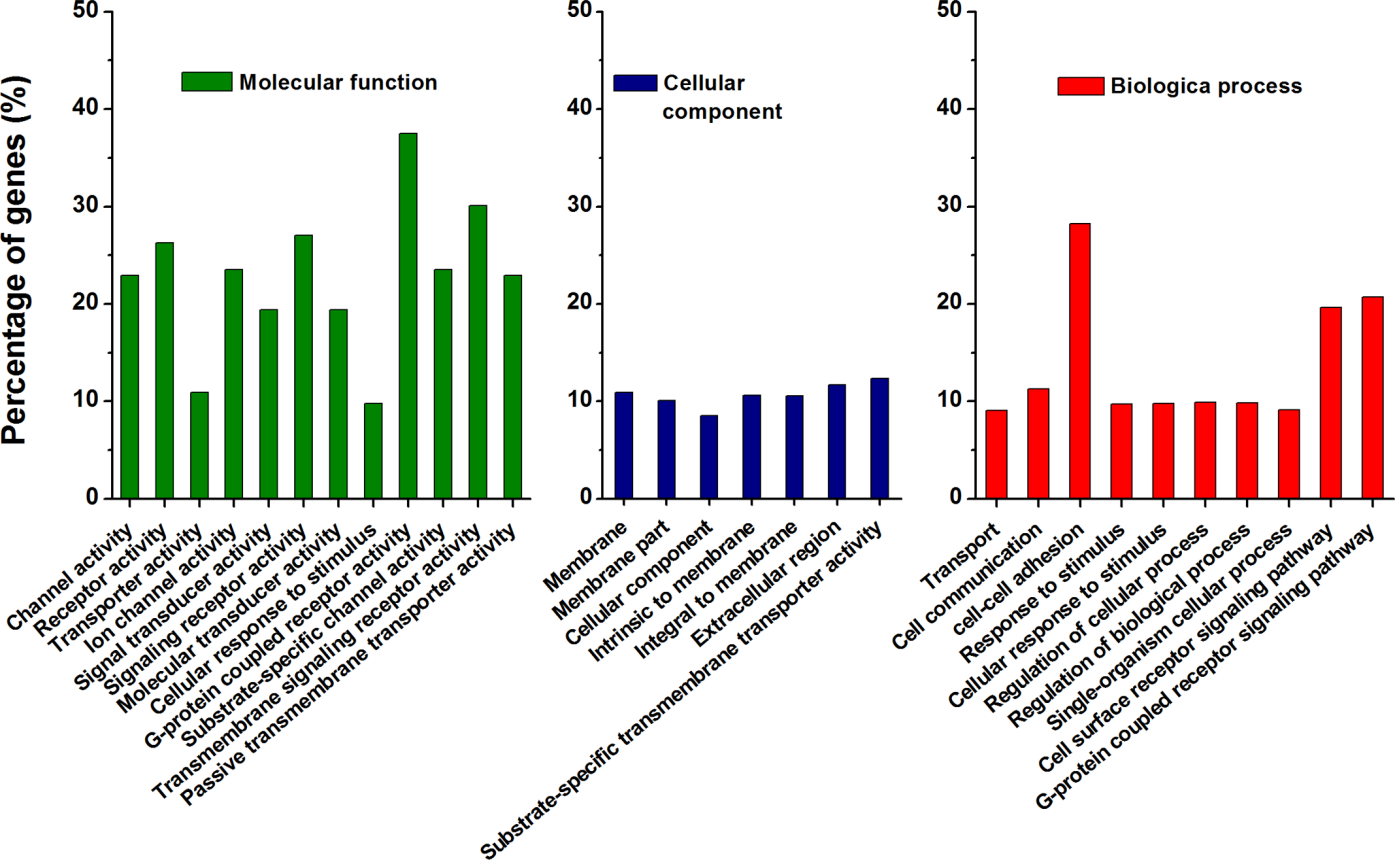
GO annotation of predicted targets for the 807 differentially expressed genes between the cultured vesicles and protoscoleces. The top 29 clusters are shown. The x-axis displays the name of the GO term, and the y-axis corresponds to the percentage of the differentially expressed genes in the term. Different pillar colors in the histogram are used to distinguish between different biological processes.

To investigate the functional associations of the 807 differentially expressed genes, we performed GO analysis using the GO database (Fig 8, S3 and S4 Tables). The results demonstrated that the up-regulated differentially expressed genes were enriched for, among others, cyclic nucleotide biosynthetic and metabolic process, gluconate and aldonate transmembrane transport, anion transport, phosphorus−oxygen lyase activity, sulfuric ester hydrolase activity and lyase activity. The down-regulated differentially expressed genes were enriched for, among others, response to signaling receptor activity, regulation of cellular process, regulation of biological process, stimulus, homophilic cell adhesion, cell communication process, transmembrane signaling receptor activity, ion transmembrane and passive transmembrane transporter activity. We also performed KEGG pathway analysis on the differentially expressed genes (S6 Fig, S5 and S6 Tables). A significant molecular function involving a neuroactive ligand-receptor interaction was enriched. Furthermore, we randomly selected 40 differentially expressed genes (S7 Table) might involve in the enriched KEGG pathways (S7 and S8 Figs) to perform expression pattern validation using q-PCR. For example, Rho GTP binding protein RhoG and Secreted frizzled protein gene might involve in Wnt signaling pathway (S5 and S6 Tables). For these selected genes, the correlation between the mRNA expression level from q-PCR and RNA-seq was relatively high, confirming the high reproducibility of the RNA-seq data in this study (S7, S8 and S9 Figs).

## Discussion

Disease models based on three-dimensional cell culture have advanced our understanding of the molecular and cellular mechanisms that underlie pathogenic processes in both the host and pathogen. They have enabled the study of difficult-to-culture pathogens, and have exciting potential for use as powerful screening tools for therapeutics and for drug and vaccine target discovery and validation [21]. Thus, in this study, we established a 3D *in vitro* cultivation system of a key part of the *E*. *multilocularis* life cycle, involving the development of protoscoleces to metacestode vesicles (protoscoleces de-differentiated towards the metacestode). We used a 3D co-culturing approach by seeding freshly isolated hepatocytes with MSCs (at a ratio of 5:1) in a 3D collagen scaffold. The co-culture system sustained the hallmarks of hepatocyte-specific functions as evidenced by albumin and urea product ion, EROD and CYP450 enzyme activity, and gene expression analyses. There are two important reasons for the successful development of this hepatic organoid. First, the 3D porous scaffold that is made from collagen allows the hepatocytes and MSCs to form a microenvironment conducive to cellular proliferation and maturation. Collagen is the major component of the ECM and it has been widely utilized to culture cells because of its excellent characteristics, including biocompatibility, mechanical strength, degradability and limited immunogenicity [33]. 3D matrices composed of natural collagen [34], synthetic biomaterials [35], hydrogel [36], microfluidic device [37], and rotating wall vessel[30] have been used for hepatocyte culture. We used a novel porous 3D collagen scaffold, which has been successfully used in cancer stem cell studies [38]. Moreover, our culture system is an original 3D co-culture system of hepatocytes and MSC based on the novel collagen scaffold. Second, the co-culture of hepatocytes with MSCs enhanced hepatocyte phenotypic and functional maintenance. MSCs are relatively easy to isolate, expand rapidly for 30 or more doublings in culture, and differentiate into several cellular phenotypes *in vitro* and *in vi*vo [39]. MSCs can secrete several growth factors and cytokines, such as HGF, epidermal growth factor, IL-6, and TNF-a, to stimulate hepatocyte proliferation, reduce hepatocyte apoptosis, and enhance hepatocyte functionality, as indicated by the high levels of albumin and urea secretion under 2D conditions [40–44].

This successful development of 3D hepatic organoids is the foundation for *E*. *multilocularis* studies, and to our knowledge, this is the first time it has been applied to study the development of *E*. *multilocularis* larvae. In addition, unlike previously reported 3D hepatocyte culture systems based on collagen hydrogels [36] or a collagen sandwich [45], this 3D hepatic model was more amenable for *E*. *multilocularis* culture. Here, a protoscolex de-differentiated into a hydatid vesicles either by the growth of a small posterior bladder or by the swelling of the protoscolex. As the culture time increased, the young micro vesicles quickly increased in size and became typically unilocular and spherical in shape. The maximum size of the vesicles reached approximately 9.06 mm. Furthermore, the number of protoscoleces that underwent de-differentiation was high (about 81.2% of all protoscoleces in culture).

These results suggest that the 3D co-culture system provided the nutritional needs and the production of certain soluble low-molecular-weight growth factors [9] required for inducing the de-differentiation of protoscoleces and the long-term growth of the metacestodes. Further, the culture media added a variety of supplements required for the culture of hepatocytes that were also necessary for metacestode development. In particular, the presence of insulin in the culture media helped stimulate the formation of metacestode vesicles from protoscoleces [46,47], and EGF promoted the germinative cell proliferation of vesicles [48].

However, the production of multiple protoscoleces (initially collected in Yushu, Qinghai province) by asexual division in these vesicles was not achieved. This might be because of the down-regulation of Wnt signaling pathway, which related to the asexual budding of scoleces [49]. More interestingly, we also co-cultured *E*. *multilocularis* protoscoleces from different collected regions (originally isolated from a naturally infected plateau pika from Jiuzhi County, Qinghai province, China), developed as cysts and then produced multiple protoscoleces (S5 Fig). This might be because of the increased viability of the different original *E*. *multilocularis* isolate and this is the subject of future study.

Moreover, these vesicles could have easily been broken or had collapsed. Even the fluid flow of medium replacement could cause the damage of the vesicles as the vesicles lacked the host collagen capsule (adventitial layer), which surrounds the laminated layer as a pericyst with a fibrous adventitial layer produced by the resolution of the precociously initiated host cellular inflammation *in vivo* [50,51]. The likely lack of the host component adventitial layer made it possible, however, to investigate metacestode differentiation at the molecular level. We were able to show by transcriptome sequencing mapping rates (more than 86%) that the vesicles were likely free from host contamination (S1 Text and S8 Table). Gene ontology and pathway analysis indicated that, compared with the protoscoleces, the vesicles appeared to have higher metabolic activity to adapt to rapid cell proliferation which in turn caused their size to increase rapidly. The down-regulation of Neuroactive ligand-receptor interaction category in the vesicle might correlate with the gradual decrease of movement in the vesicularised protoscolex and the complete loss of movement when the cyst was finally formed [1,52]. Further, the down regulation of the response to signaling receptor activity, regulation of stimulus, homophilic cell adhesion, cell communication process, transmembrane signaling receptor activity, ion transmembrane and passive transmembrane transporter activity might lead to a reduction in the parasite response to external stimulation. These results provided important information as the genes uniquely expressed in protoscoleces or vesicles might be key to understanding the development of *E*. *multilocularis* larvae, a process we will focus on in the future.

In general, in combination with 3D co-cultured hepatocytes and MSCs on 3D collagen scaffolds, the system we describe closely simulates a native liver and constitutes a useful tool to mimic the formation of metastases during chronic *E*. *multilocularis* infections, as well as the events that occur during the development/differentiation of a protoscolex into a hydatid cyst. To our knowledge, this is the first time this 3D hepatic model has been applied to study the prolonged *in vitro* cultivation of *E*. *multilocularis* protoscoleces. We anticipate that this 3D model will prove useful in evaluating potential therapeutic strategies for AE and in further exploration of the molecular mechanisms governing of *E*. *multilocularis* larval development.

## Supporting information

S1 Text Supplementary materials and methods. (PDF)

S1 Fig Photograph of the actual collagen scaffolds. (A) The collagen scaffolds are circular wafers with uniform size. (B) The diameter of the collagen scaffold is about 5 mm.
(PDF)

S2 Fig Light microscopy images of hepatocytes (A-D) and MSCs (G-J) cultured for 3, 5, 7 and 10 days in 2D cultured condition. Hepatocytes (10 d in vitro) are immunostained with the marker protein CK19 and the nuclear Hoechst dye (E, F). MSCs (10 d in vitro) are immunostained with the CD44 and the nuclear Hoechst dye (K, L). Scale bar: 20 μm.
(PDF)

S3 Fig Viability assay of protoscoleces. Trypan blue-staining image of protoscoleces. The protoscoleces with no absorbed dye were considered potentially viable and otherwise, they were recorded as dead. Scale bar: 100 μm.
(PDF)

S4 Fig Photographs and SEM images of the collected vesicles used for total RNA extraction. (A) The vesicles were cultured for eight weeks and reached a diameter of more than 5 mm. (B) Image B is the higher magnification image of the boxed area in A. Scale bar: 5 mm. (C) SEM image of the internal surface (laminated layer) of the vesicle. (D) Image D is the higher magnification image of the boxed area in C.
(PDF)

S5 Fig Microscopyical images of cultured protoscoleces and vesicles. Light microscopical images of invaginated protoscoleces (A), evaginated protoscoleces (B), dead vesicles (C), healthy vesicles (D), protoscoleces that have commenced differentiation (E) and develops within the brood capsules (F). Images E’ and F’ are the higher magnification images of the boxed areas in E and F, respectively. Scale bar: 100 μm.
(PDF)

S6 Fig Scatterplot of KEGG pathway analysis of the differentially expressed genes between the vesicles and protoscoleces. (A) Twenty of the 642 down regulated genes in the enrichment pathway. (B) Twenty of the 165 up regulated genes in the enrichment pathway. The y-axis displays the name of the pathway, and the x-axis indicates the rich factor. The size of the points corresponds to the number of differentially expressed genes in the pathway. Different colors of the points indicate a different q-value.
(PDF)

S7 Fig Quantitative PCR analysis of 20 down regulated genes in the vesicles compared with the PSCs. The y-axis displays 2^-(∆∆Ct)^ of the genes. Gene systematic name (gene ID) is marked in each histogram.
(PDF)

S8 Fig Quantitative PCR analysis of 20 up regulated genes in vesicles compared with the PSCs. The y-axis displays 2^-(∆∆Ct)^ of the genes. Gene systematic name (gene ID) is marked in each histogram.
(PDF)

S9 Fig RNA-seq fold change of the 20 down regulated genes and 20 up regulated genes in vesicles compared with the PSCs. The gene systematic name (gene ID) is marked in each histogram.
(PDF)

S10 Fig PCR amplified *nad*1 (A) and *cox*1 (B) fragments examined in 1% (w/v) agarose gels stained with ethidium bromide. Lane M, DL2000 molecular marker; Lane S, *E*. *multilocularis* metacestodes.
(PDF)

S1 Table The details of differentially expressed genes between cultured vesicles and protoscoleces. (XLSX)

S2 Table Unique genes expressed in protoscoleces or vesicles. (XLSX)

S3 Table The details of gene ontology enrich result of down regulated genes. (XLSX)

S4 Table Details of gene ontology enrichment result of up regulated genes. (XLSX)

S5 Table The details of KEGG pathway enrich result of down regulated genes. (XLSX)

S6 Table The details of KEGG pathway enrichment result of up regulated genes. (XLSX)

S7 Table Information and primer details of the 2 house-keeping genes and the 40 randomly selected genes. (XLSX)

S8 Table Summary of RNA-seq read mapping. (XLSX)
