# Supplementary material for: Three-dimensional hepatocyte culture system for the study of Echinococcus multilocularis larval development
Source: PLoS Negl Trop Dis. 2018 Mar 14;12(3):e0006309. doi: 10.1371/journal.pntd.0006309 (PMC5868855; doi:10.1371/journal.pntd.0006309)
Supplement: S1 Text — (PDF) [file pntd.0006309.s001.pdf]

Three-dimensional Hepatocyte Culture System for the Study of *Echinococcus multilocularis* Larval Development

Li Li[1], Bing Chen[2], Hongbin Yan[1], Yannan Zhao[2], Zhongzi Lou[1], Jianqiu Li[1], Baoquan Fu[1], Xingquan Zhu[1], Donald P. McManus[3], Jianwu Dai[2*], Wanzhong Jia[1*]

[1]State Key Laboratory of Veterinary Etiological Biology, Key Laboratory of Veterinary Parasitology of Gansu Province, Key Laboratory of Veterinary Public Health of Agriculture Ministry, Lanzhou Veterinary Research Institute, Chinese Academy of Agricultural Sciences, Lanzhou, Gansu 730046, P. R. China.

[2]State Key Laboratory of Molecular Developmental Biology, Institute of Genetics and Developmental Biology, Chinese Academy of Sciences, Beijing 100190, P. R. China.

[3]Molecular Parasitology Laboratory, QIMR Berghofer Medical Research Institute, Brisbane, QLD 4006, Australia.

[*]Correspondence authors:

Jian-Wu Dai, jwdai@genetics.ac.cn; Tel. +86-010-82614426, Fax. +86-010-82614426

Wan-Zhong Jia, jiawanzhong@caas.cn; Tel. +86-0931-8312212, Fax. +86-0931-8340977

S1 Supplementary materials and methods.

Cell assay

To prepare the SEM sample, samples were washed with cold D-PBS three times and fixed with a cold fixative (3% glutaraldehyde + 2% paraformaldehyde in 0.1 M phosphate buffer) for 30 min at 4 ℃. The samples were dehydrated in an ethanol series (50%, 75%, 85%, 95%, 100%, and 100%). Samples were critically point-dried and gold-coated with a sputter-coater at 15 mA for 60

s. Then, the samples were visualized with the SEM.

Cell RNA isolation and semi-quantitative RT-PCR

The synthetic oligonucleotide primer sequences were as follows: CLDN-3, 5'-TCA TCG TGG TGT CCA TCC TG-3' and 5'-CGT AGT CCT TGC GGT CGT AG-3'; Bsep, 5'-AGA AGA GGC GAC AAT GGA-3' and 5'-TGA TAG GCG ATG GGC AAC-3'; AFP, 5'-CGT TAG ATT CCT CCC AGT G-3' and 5'-TTC AGG TTT GAC GCC ATT-3'; G6P, 5'-AAT CTC CTC TGG GTG GCA-3' and 5'-GCA TGG CGG TTG ACT TTA-3'; A1AT, 5'-GCA GCA TCT GGA GCA AAC-3' and 5'-CAT CGT AGG GTG GTC ATT-3'; GAPDH, 5'-TCA ACG GCA CAG TCA AGG -3' and 5'-AAG TCG CAG GAG ACA ACC-3'; CYP3A4, 5'- CTG TCA GCC TGG TGC TCC TCT ATC -3' and 5'- TGC TGG ACA TCA GGG TGA GTG GC -3'; NR1I3, 5'-CCATTGGTCCCATCTGTCCG -3' and 5'- AACCGACTTTGGAGCCGAGA -3'. The predicted sizes of the amplified Cldn-3, Bsep, AFP, G6P, A1AT, GAPDH, CYP3A4, and NR1I3 DNA products were 409, 295, 918, 937, 282, 691, 1062 and 712 bp, respectively.

Total RNA extraction was carried out using an RNeasy kit, and the reverse transcripts were synthesized using a RevertAid^{TM} First Strand cDNA Synthesis kit. Briefly, the total RNA of each disposal was extracted with a guanidinium isothiocyanate extraction followed by selectively binding to a silica-based membrane. The extracted RNA samples were dissolved in DI water treated with 0.1% diethylpyrocarbonate and stored at -80 ℃ until further use. cDNA synthesis was performed using M-MuLV reverse transcriptase. For PCR amplification, in each 20 μL reaction mixture, 2 μL of cDNA solution was used with one unit of *Taq* DNA polymerase and 10 pmol of each specific primer diluted in PCR buffer supplemented with 1.5 mM $MgCl_2$. The thermal cycling conditions were as follows: 94 ℃ for 3 min, followed by 35 cycles of 94 ℃ for 20 s, 55°C for 1 min, and 72°C for 1 min, and a final extension at 72°C for 7 min. The PCR

products were analyzed by standard agarose gel electrophoresis, stained with ethidium bromide, visualized using a UV trans-illuminator and photographed using Peiqing TS-680B gel documentation systems (Peiqing Science Technology Co., Ltd, Shanghai, China). The photographs were scanned, and the electrophoresis gel images were analyzed.

E. multilocularis infected mouse model molecular identification

Vesicles DNA was extracted using QIAmp DNA FFPE Tissue Kit (Qiagen, US). Molecular identification was undertaken by PCR-amplifying the complete fragments of the mitochondrial cytochrome c oridase subunit 1 (*cox*1) gene and NADH dehydrogenase subunit 1 (*nad*1) gene. The primers were designed based on the multiple sequence alignment of *E. multilocularis* (whose intermediate hosts include rodents) complete mt genomes deposited in the National Center for Biotechnology Information (NCBI) GenBank (*nad*1-F: 5’-GAG TTT GCG TCT CGA TGA TAG G’-3, *nad*1-R: 5’-TCC CCA AAA CCC ACA TTC TAC-3’; *cox*1-F: 5’-AGG TTT GAC TTT CTC TTT GGT T-3’, *cox*1-R: 5’-CCA ACA AAT CCA AAT AAA CGG-3’). PCR products were purified directly from an agarose gel (1%) using an Axy Prep™ DNA Gel Extraction kit (AXVGEN, USA) and then sent to a commercial company (Genewiz Biotech, Beijing, China) for sequencing. The sequences were submitted to NCBI GenBank, to search for homologous sequences using Basic Local Alignment Search Tools (BLAST), and then deposited in NCBI GenBank.

Preparation of protoscoleces

To isolate the protoscoleces, parasite tissue was first isolated from infected mice and homogenized [1]. The homogenate was subsequently filtered once through a 150-μm pore size nylon mesh, thus separating the protoscoleces from large pieces of metacestode tissue. The flow through was filtered through a 50-μm pore size nylon mesh, separating the protoscoleces from

single cells and small cell clumps. Protoscoleces were then washed off the nylon mesh with sterile PBS and manually separated from equal sized vesicles using a pipette tip under a microscope.

Parasite RNA-seq

To select cDNA fragments that were preferentially 150-200 bp in length, the library fragments were purified with an AMPure XP system (Beckman Coulter, Beverly, USA). Finally, PCR products were purified (AMPure XP system) and the library quality was assessed on the Agilent Bioanalyzer 2100 system. The clustering of the index-coded samples was performed on a cBot Cluster Generation System using the TruSeq PE Cluster Kit v3-cBot-HS (Illumina). After cluster generation, the library preparations were sequenced on an Illumina Hiseq 2000 platform and 100 bp paired-end reads were generated. Raw data (raw reads) in fast q format were first processed through in-house Perl scripts. We mapped the RNA-Seq reads to *E. multilocularis* genome (ftp://ftp.sanger.ac.uk/pub/pathogens/Echinococcus/multilocularis/genome/Emultilocularis_genome_v3.fas and http://www.genedb.org/Homepage/Emultilocularis) by using TopHat [2].

Real-Time PCR and statistical analysis

TRIzol reagent was used to extract the total RNA from these samples. An AMV First Strand cDNA Synthesis Kit was used to synthesize the first-strand cDNA. Primer Premier 5.0 was used to design gene-specific primers, which were validated using Oligo 6.0. The primer sequences are shown in Table S7. The mRNA levels of the differentially expressed genes were normalized against two housekeeping genes, *E. multilocularis* GAPDH and the actin gene, in the corresponding samples. qRT-PCR was carried out in triplicate with SybrGreen PCR Master Mix (2X, ABI, USA) on a Stepone Real-Time PCR System (Stepone plus, ABI, USA) and performed in a 20 μL reaction unit containing 2 μL of cDNA as a template with each specific

oligonucleotide primer pair using the following program: 95 ℃ for 3 min, 40 cycles of 95 ℃ for 15 s, 60 ℃ for 40 s, 57-60 ℃ for 30 s and 72 ℃ for 6 min. Data were expressed as the mean ± S.D. Pairwise comparisons were performed Using Student's t test.

References

1. Jura H, Bader A, Hartmann M, Maschek H, Frosch M. Hepatic tissue culture model for study of host-parasite interaction in alveolar echinococcosis. Infect Immun. 1996; **64**: 3484–3490. PMID: 8751888

2. Trapnell C, Pachter L, Salzberg SL. TopHat: discovering splice junctions with RNA-Seq. Bioinformatics. 2009; 25(9): 1105–1111. https://doi.org/10.1093/bioinformatics/btp120 PMID: 19289445
