# Supplementary material for: Three-dimensional hepatocyte culture system for the study of Echinococcus multilocularis larval development
Source: PLoS Negl Trop Dis. 2018 Mar 14;12(3):e0006309. doi: 10.1371/journal.pntd.0006309 (PMC5868855; doi:10.1371/journal.pntd.0006309)
Supplement: S1 Fig — (A) The collagen scaffolds are circular wafers with uniform size. (B) The diameter of the collagen scaffold is about 5 mm. (PDF) [file pntd.0006309.s002.pdf]

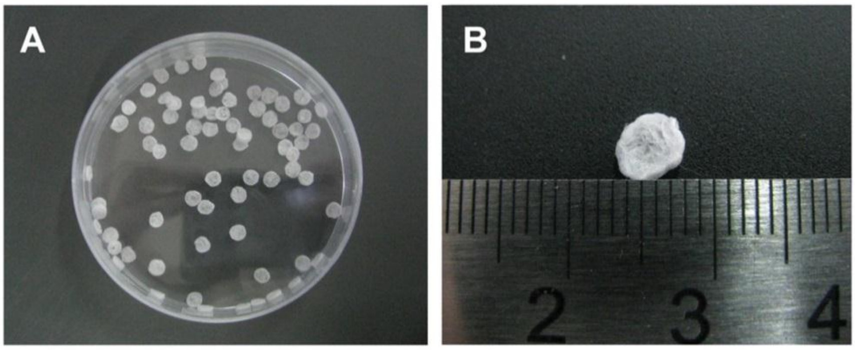

S1 Fig. Photograph of the actual collagen scaffolds. (A) The collagen scaffolds are circular wafers with uniform size. (B) The diameter of the collagen scaffold is about 5 mm.
