# Supplementary material for: Three-dimensional hepatocyte culture system for the study of Echinococcus multilocularis larval development
Source: PLoS Negl Trop Dis. 2018 Mar 14;12(3):e0006309. doi: 10.1371/journal.pntd.0006309 (PMC5868855; doi:10.1371/journal.pntd.0006309)
Supplement: S2 Fig — Light microscopy images of hepatocytes (A-D) and MSCs (G-J) cultured for 3, 5, 7 and 10 days in 2D cultured condition. Hepatocytes (10 d in vitro) are immunostained with the marker protein CK19 and the nuclear Hoechst dye (E, F). MSCs (10 d in vitro) are immunostained with the CD44 and the nuclear Hoechst dye (K, L). Scale bar: 20 μm. (PDF) [file pntd.0006309.s003.pdf]

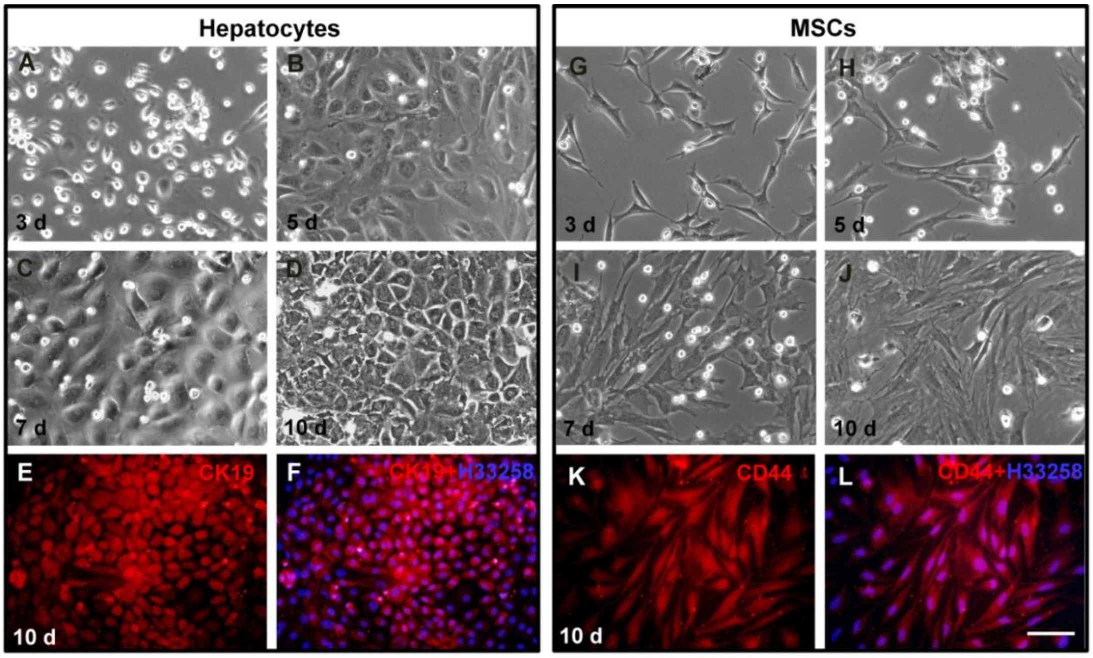


**S2 Fig.** Light microscopy images of hepatocytes (A-D) and MSCs (G-J) cultured for 3, 5, 7 and 10 days in 2D cultured condition. Hepatocytes (10 d in vitro) are immunostained with the marker protein CK19 and the nuclear Hoechst dye (E, F). MSCs (10 d in vitro) are immunostained with the CD44 and the nuclear Hoechst dye (K, L). Scale bar: 20 μm.
