# Supplementary material for: Three-dimensional hepatocyte culture system for the study of Echinococcus multilocularis larval development
Source: PLoS Negl Trop Dis. 2018 Mar 14;12(3):e0006309. doi: 10.1371/journal.pntd.0006309 (PMC5868855; doi:10.1371/journal.pntd.0006309)
Supplement: S3 Fig — Trypan blue-staining image of protoscoleces. The protoscoleces with no absorbed dye were considered potentially viable and otherwise, they were recorded as dead. Scale bar: 100 μm. (PDF) [file pntd.0006309.s004.pdf]

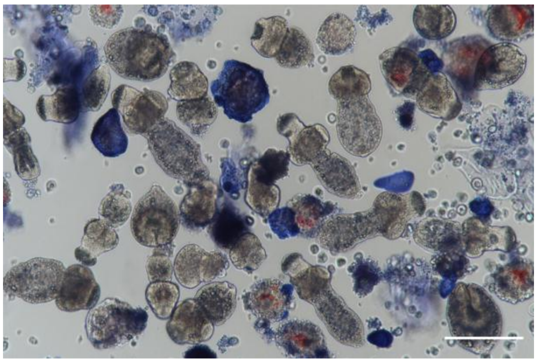

S3 Fig. Viability assay of protoscolices. Trypan blue-staining image of protoscolices. The protoscolices with no absorbed dye were considered potentially viable and otherwise, they were recorded as dead. Scale bar: 100 μm.
