# Supplementary material for: Three-dimensional hepatocyte culture system for the study of Echinococcus multilocularis larval development
Source: PLoS Negl Trop Dis. 2018 Mar 14;12(3):e0006309. doi: 10.1371/journal.pntd.0006309 (PMC5868855; doi:10.1371/journal.pntd.0006309)
Supplement: S4 Fig — (A) The vesicles were cultured for eight weeks and reached a diameter of more than 5 mm. (B) Image B is the higher magnification image of the boxed area in A. Scale bar: 5 mm. (C) SEM image of the internal surface (laminated layer) of the vesicle. (D) Image D is the higher magnification image of the boxed area in C. (PDF) [file pntd.0006309.s005.pdf]

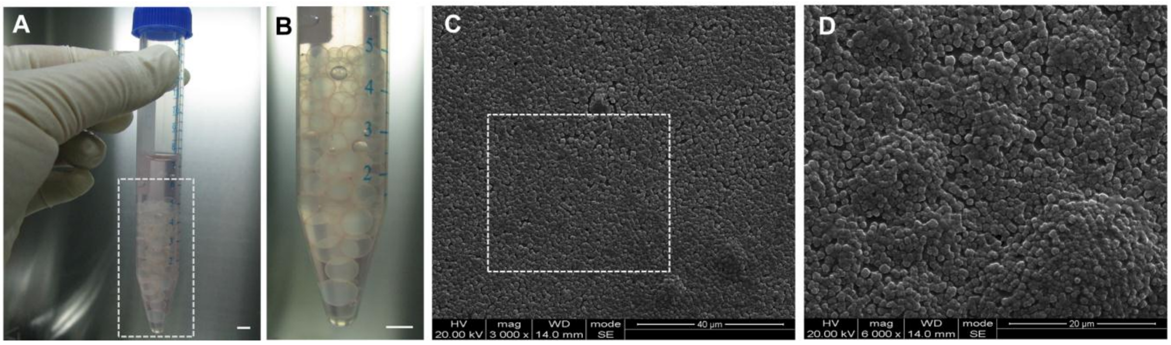


**S4 Fig**. Photographs and SEM images of the collected vesicles used for total RNA extraction. (A) The vesicles were cultured for eight weeks and reached a diameter of more than 5 mm. (B) Image B is the higher magnification image of the boxed area in A. Scale bar: 5 mm. (C) SEM image of the internal surface (laminated layer ) of the vesicle. (D) Image D is the higher magnification image of the boxed area in C.
