# Supplementary material for: Three-dimensional hepatocyte culture system for the study of Echinococcus multilocularis larval development
Source: PLoS Negl Trop Dis. 2018 Mar 14;12(3):e0006309. doi: 10.1371/journal.pntd.0006309 (PMC5868855; doi:10.1371/journal.pntd.0006309)
Supplement: S5 Fig — Light microscopical images of invaginated protoscoleces (A), evaginated protoscoleces (B), dead vesicles (C), healthy vesicles (D), protoscoleces that have commenced differentiation (E) and develops within the brood capsules (F). Images E’ and F’ are the higher magnification images of the boxed areas in E and F, respectively. Scale bar: 100 μm. (PDF) [file pntd.0006309.s006.pdf]

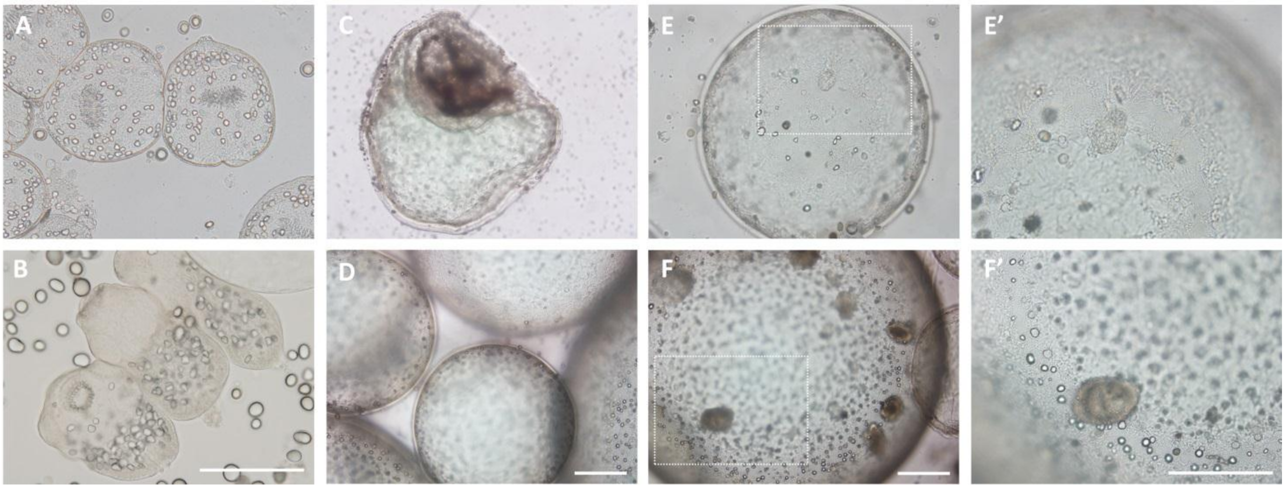

S5 Fig. Microscopic images of cultured protoscoleces and vesicles. Light microscopical images of invaginated protoscoleces (A), evaginated protoscoleces (B), dead vesicles (C), healthy vesicles (D), protoscoleces that have commenced differentiation (E) and develops within the brood capsules (F). Images E' and F' are the higher magnification images of the boxed areas in E and F, respectively. Scale bar: 100 μm.
