# Supplementary material for: Three-dimensional hepatocyte culture system for the study of Echinococcus multilocularis larval development
Source: PLoS Negl Trop Dis. 2018 Mar 14;12(3):e0006309. doi: 10.1371/journal.pntd.0006309 (PMC5868855; doi:10.1371/journal.pntd.0006309)
Supplement: S6 Fig — (A) Twenty of the 642 down regulated genes in the enrichment pathway. (B) Twenty of the 165 up regulated genes in the enrichment pathway. The y-axis displays the name of the pathway, and the x-axis indicates the rich factor. The size of the points corresponds to the number of differentially expressed genes in the pathway. Different colors of the points indicate a different q-value. (PDF) [file pntd.0006309.s007.pdf]

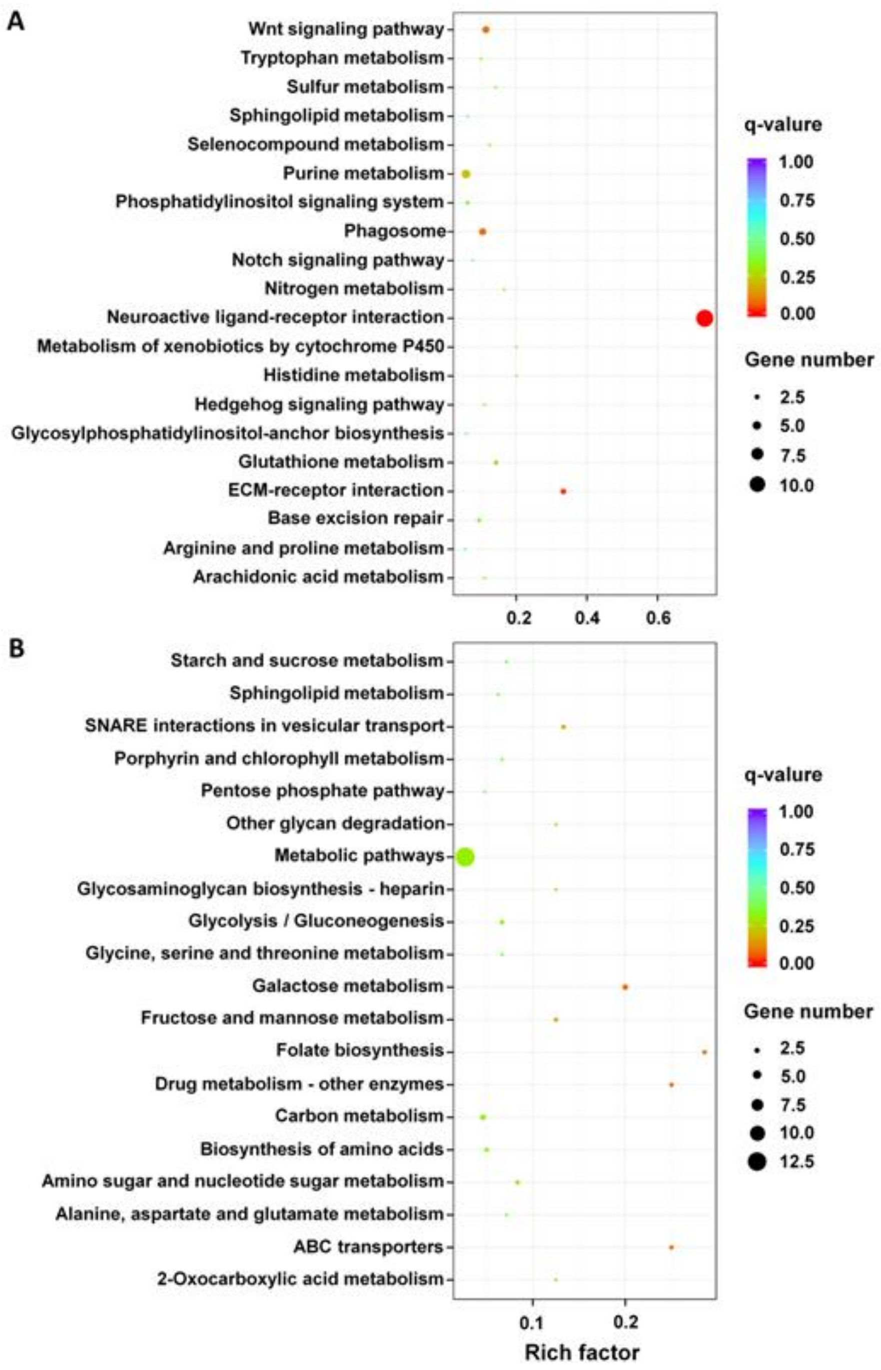

S6 Fig. Scatterplot of KEGG pathway analysis of the differentially expressed genes between the vesicles and protoscoleces. (A) Twenty of the 642 down regulated genes in the enrichment pathway. (B) Twenty of the 165 up regulated genes in the enrichment pathway. The y-axis displays the name of the pathway, and the x-axis indicates the rich factor. The size of the points corresponds to the number of differentially expressed genes in the pathway. Different colors of the points indicate a different q-value.
