# Supplementary material for: Three-dimensional hepatocyte culture system for the study of Echinococcus multilocularis larval development
Source: PLoS Negl Trop Dis. 2018 Mar 14;12(3):e0006309. doi: 10.1371/journal.pntd.0006309 (PMC5868855; doi:10.1371/journal.pntd.0006309)
Supplement: S8 Fig — The y-axis displays 2-(∆∆Ct) of the genes. Gene systematic name (gene ID) is marked in each histogram. (PDF) [file pntd.0006309.s009.pdf]

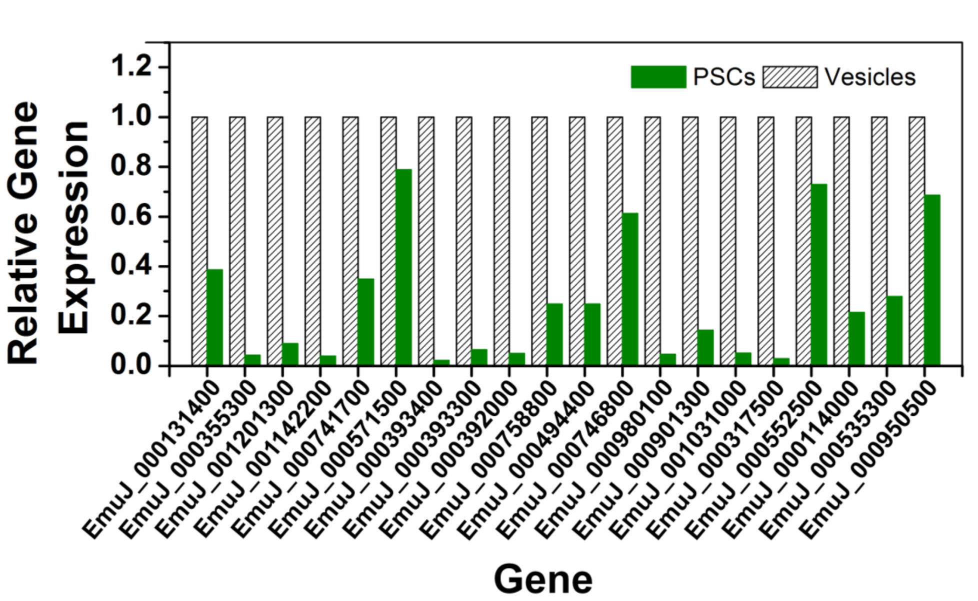

S8 Fig. Quantitative PCR analysis of 20 up regulated genes in vesicles compared with the PSCs. The y-axis displays $2^{-(\Delta\Delta Ct)}$ of the genes. Gene systematic name (gene ID) is marked in each histogram.
