# Supplementary material for: Three-dimensional hepatocyte culture system for the study of Echinococcus multilocularis larval development
Source: PLoS Negl Trop Dis. 2018 Mar 14;12(3):e0006309. doi: 10.1371/journal.pntd.0006309 (PMC5868855; doi:10.1371/journal.pntd.0006309)
Supplement: S9 Fig — The gene systematic name (gene ID) is marked in each histogram. (PDF) [file pntd.0006309.s010.pdf]

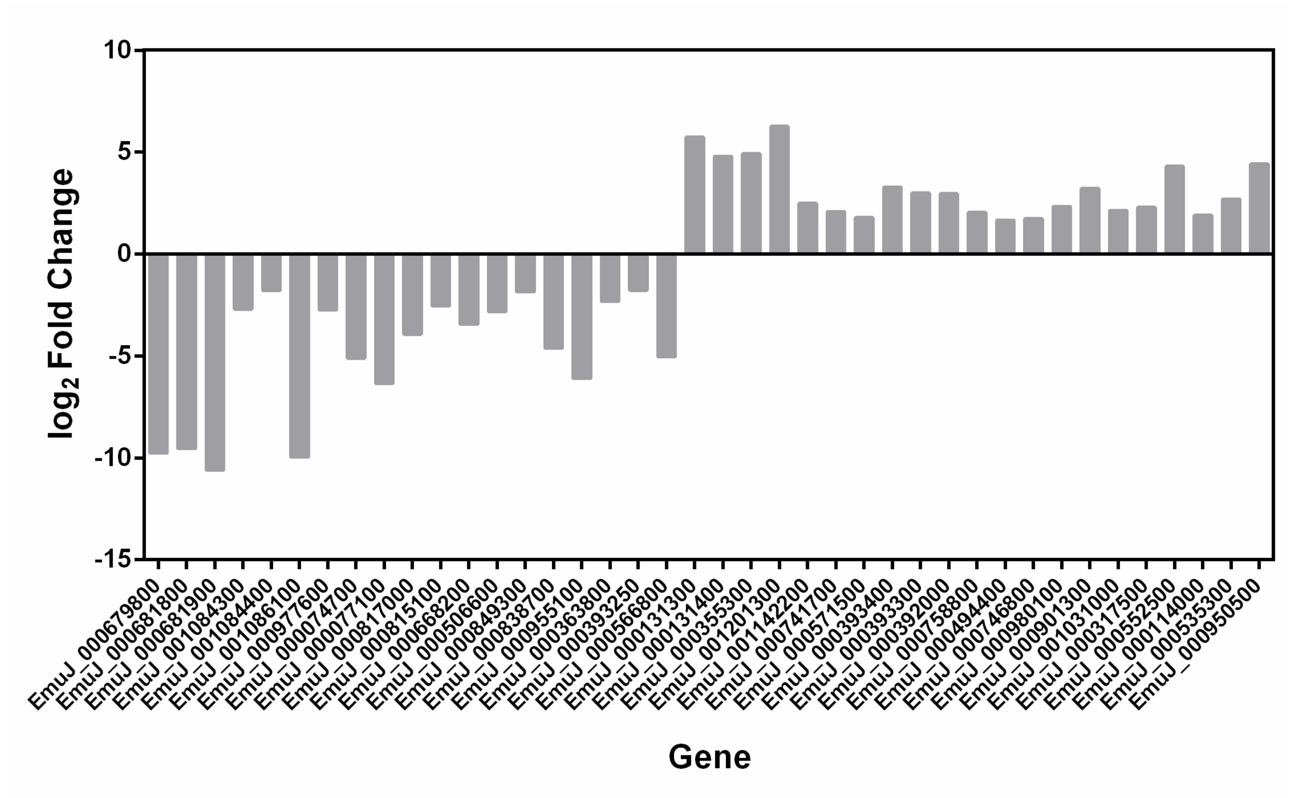

S9 Fig. RNA-seq fold change of the 20 down regulated genes and 20 up regulated genes in vesicles compared with the PSCs. The gene systematic name (gene ID) is marked in each histogram.
