# Supplementary material for: Three-dimensional hepatocyte culture system for the study of Echinococcus multilocularis larval development
Source: PLoS Negl Trop Dis. 2018 Mar 14;12(3):e0006309. doi: 10.1371/journal.pntd.0006309 (PMC5868855; doi:10.1371/journal.pntd.0006309)
Supplement: S10 Fig — PCR amplified nad1 (A) and cox1 (B) fragments examined in 1% (w/v) agarose gels stained with ethidium bromide. Lane M, DL2000 molecular marker; Lane S, E. multilocularis metacestodes. (PDF) [file pntd.0006309.s011.pdf]

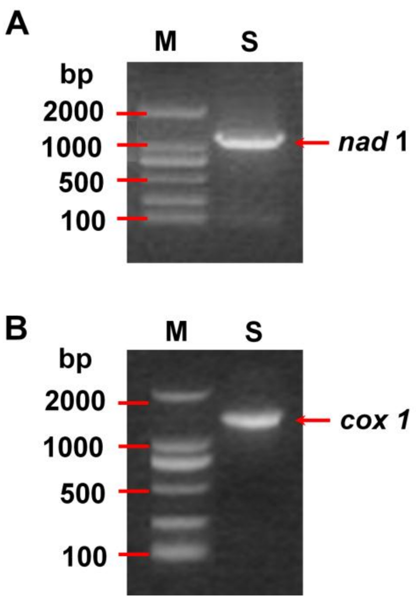

S10 Fig. PCR amplified *nad*1 (A) and *cox*1 (B) fragments examined in 1% (w/v) agarose gels stained with ethidium bromide: Lane M, DL2000 molecular marker; Lane S, *E. multilocularis* metacestodes.
